# Supplementary material for: Exploring New Routes for Genetic Resistances to Potyviruses: The Case of the Arabidopsis thaliana Phosphoglycerates Kinases (PGK) Metabolic Enzymes
Source: Viruses. 2022 Jun 8;14(6):1245. doi: 10.3390/v14061245 (PMC9228606; doi:10.3390/v14061245)
Supplement: Supplementary file 1 [file viruses-14-01245-s001.zip › SD Figure S1.pdf]

```
Alignment: C:\Users\mmdiop\Desktop\Arbre III-17-11-2021\Séquences PGKs utilisées\Afaire avec liste de PGKs restreint\al
Seaview [blocks=10 fontsize=8 A4] on Fri Dec 10 12:33:36 2021

            1                                                            Met1
Zm00001d04  ---------- ---------- ---------- ---------- ---------- ------MASA LVPASFSLAH PAARRPVNST GPA---AMLM
AQK68740.1  ---------- ---------- ---------- ---------- ---------- ---------- ---------- ---------- ----------
At_PGK3     ---------- ---------- ---------- ---------- ---------- ---------- ---------- ---------- ----------
Alyrata_PG  ---------- ---------- ---------- ---------- ---------- ---------- ---------- ---------- ----------
XP_0091066  ---------- ---------- ---------- ---------- ---------- ---------- ---------- ---------- ----------
XP_0137269  ---------- ---------- ---------- ---------- ---------- ---------- ---------- ---------- ----------
XP_0225516  ---------- ---------- ---------- ---------- ---------- ---------- ---------- ---------- ----------
XP_0091283  ---------- ---------- ---------- ---------- ---------- ---------- ---------- ---------- ----------
XP_0084470  ---------- ---------- ---------- ---------- ---------- ---------- ---------- ---------- ----------
NP_0013123  ---------- ---------- ---------- ---------- ---------- ---------- ---------- ---------- ----------
NP_0012754  ---------- ---------- ---------- ---------- ---------- ---------- ---------- ---------- ----------
NP_0013165  ---------- ---------- ---------- ---------- ---------- ---------- ---------- ---------- ----------
XP_0219934  ---------- ---------- ---------- ---------- ---------- ---------- ---------- ---------- ----------
XP_0220396  ---------- ---------- ---------- ---------- ---------- ---------- ---------- ---------- ----------
at_cPGK2    ---------- ---------- ---------- ---------- ---------- ------MAST AATAALSIIK STGGAA---V TRS---SRAS
Alyrata_cP  ---------- ---------- ---------- ---------- ---------- ------MAST AATAALSIIK STGGAA---V TRS---SRAS
At_cPGK1    ---------- ---------- ---------- ---------- ---------- ------MASA AASSAFSLLK STGAVA-SSA GTR---ARAS
Alyr_cPGK1  ---------- ---------- ---------- ---------- ---------- ------MASA AASPAFSLLK STGGVA-SSA GTR---ARAS
XP_0136482  ---------- ---------- ---------- ---------- ---------- ------MASV AASPAFSLLK STGGAIASST STR---ARAS
XP_0091352  ---------- ---------- ---------- ---------- ---------- ------MASV AASPAFSLLK STGGAIASSA ATR---ARAS
XP_0225670  ---------- ---------- ---------- ---------- ---------- ------MASV AASPAFSLLK STTGATASSA ATR---ARAS
XP_0136731  ---------- ---------- ---------- ---------- ---------- ------MASV AASPAFSLRK STTGAT-YSA ATR---ARAS
XP_0091246  ---------- ---------- ---------- ---------- ---------- ------MASV AASPTFSLLK STTGAT-YSA ATR---ARAS
XP_0136856  ---------- ---------- ---------- ---------- ---------- ------MSSA AASPALSLLK STGGVA-SSA ATR---ARAT
XP_0091465  ---------- ---------- ---------- ---------- ---------- ------MSTA AASPSLSLLK STGAVA-SSA ATR---ARAT
XP_0084563  ---------- ---------- ---------- ---------- ---------- ------MASA ASRATFHLLP TTSSSTSSTR SALLQFSPST
XP_0165815  ---------- ---------- ---------- ---------- ---------- ------MASA TASHTLCGIP TTSSSSSSTT NRVSPHSARF
PHT77520.1  MIEPEPQLND KKNPLNAHFQ PHSSTSSILY ISRTLCFCRT HPHTHTDKEK RKGIHTMASA TASHTLCGIP TTSSSSSSTT NRVSPHSARF
XP_0042439  ---------- ---------- ---------- ---------- ---------- ------MASA TASHTLCGIP TTSSSSTTNK STTYSSARFL
NP_0012752  ---------- ---------- ---------- ---------- ---------- ------MASA TASHTFCGIP TT-SSSSTTN RSTTHSSARF
XP_0151700  ---------- ---------- ---------- ---------- ---------- ------MASA TASHTFCGIP TTSSSSTT-N RSTTHSSARF
ADR71054.1  ---------- ---------- ---------- ---------- ---------- ------MASA TASHSLCGIP ATSSSTTNKS IAP---SSAR
XP_0164941  ---------- ---------- ---------- ---------- ---------- ------MASA TASHTLCGIP ATSSSTTNKA IAP---SSAR
XP_0220015  ---------- ---------- ---------- ---------- ---------- ------MASA PSPASLSFLP TISSSSTTTS RTPTVRLHSR
AQK86959.1  ---------- ---------- ---------- ---------- ---------- ------MASA AAPPTSLSLA ARAATRAAAA ------AAAP
Zm00001d01  ---------- ---------- ---------- ---------- ---------- ---------- ---------- ---------- ----------
A8JC04_cPG  ---------- ---------- ---------- ---------- ---------- ---------- ---MALSMKM RANARVVSGR RV----AAVA
GAQ79801.1  ---------- ---------- ---------- ---------- ---------- ---------- ---------- ---------- ----------
sp_P18912.  ---------- ---------- ---------- ---------- ---------- ---------- ---------- ---------- ----------
AAC75963.1  ---------- ---------- ---------- ---------- ---------- ---------- ---------- ---------- ----------
sp|P50315|  ---------- ---------- ---------- ---------- ---------- ---------- ---------- ---------- ----------
sp_P00560.  ---------- ---------- ---------- ---------- ---------- ------M--- ---------- ---------- ----------
NP_112467.  ---------- ---------- ---------- ---------- ---------- ------M--- ---------- ---------- ----------
sp|P07205.  ---------- ---------- ---------- ---------- ---------- ------M--- ---------- ---------- ----------
sp|P00558.  ---------- ---------- ---------- ---------- ---------- ------M--- ---------- ---------- ----------
NP_032854.  ---------- ---------- ---------- ---------- ---------- ---------- ---------- ---------- ----------
Zm00001d03  ---------- ---------- ---------- ---------- ---------- ---------- ---------- ---------- ----------
tr_A0A1S4D  ---------- ---------- ---------- ---------- ---------- ----MGQLLN PLQGIFFVIK AVAQDPAKLY RQTLSPYFSK
XP_0165427  ---------- ---------- ---------- ---------- ---------- ------MSGV LDFFLGYSLP RISDFHYGCT KRF---QDLP
XP_0063543  ---------- ---------- ---------- ---------- ---------- ---------- ---------- ---------- ----------
XP_0258888  ---------- ---------- ---------- ---------- ---------- ---MSYVLEF FLGYTLPRIS NFHNGCTKFH DLQQIRLRLS
XP_0042466  ---------- ---------- ---------- ---------- ---------- ---MSYVLEF FLGYTLPRIS NFHNGCTKFH DLQQIRLRLS
KAF3623713  ---------- ---------- ---------- ---------- ---------- ------MASA TASHTLCGIP TTSSSSSSTT NRVSPHSARF
GAQ85336.1  ---------- ---------- ---------- --------ME CTFYCSPAVL QKELSRRFSQ DQNCSGCLLS PKAAPPLHQH GTSFLRHKLH
A8JFT4_PGK  ---------- ---------- ---------- ---------- ---------- ------MQAD DPA------- ---------- ----------
```

```
                91                                                                     Met2
Zm00001d04  LRRAGLATVC PPERSLGSDC LRAGADPRLA IHVALRCRSA S-STG----- ---------- -RGTRAAATM TKKS------ ----VGDLTA
AQK68740.1  ---------- ---------- ---------- ---------- ---------- ---------- --------MA TKRS------ ----VGTLGE
At_PGK3     ---------- ---------- ---------- ---------- ---------- ---------- --------MA TKRS------ ----VGTLKE
Alyrata_PG  ---------- ---------- ---------- ---------- ---------- ---------- --------MA TKRS------ ----VGTLKE
XP_0091066  ---------- ---------- ---------- ---------- ---------- ---------- --------MA TKRS------ ----VGTLKE
XP_0137269  ---------- ---------- ---------- ---------- ---------- ---------- --------MA TKRS------ ----VGTLKE
XP_0225516  ---------- ---------- ---------- ---------- ---------- ---------- --------MA TKRS------ ----VGTLKE
XP_0091283  ---------- ---------- ---------- ---------- ---------- ---------- --------MA TKRS------ ----VGTLKE
XP_0084470  ---------- ---------- ---------- ---------- ---------- ---------- --------MA TKRS------ ----VGTLKE
NP_0013123  ---------- ---------- ---------- ---------- ---------- ---------- --------MA VKKS------ ----VGSLKE
NP_0012754  ---------- ---------- ---------- ---------- ---------- ---------- --------MA VKKS------ ----VGSLKE
NP_0013165  ---------- ---------- ---------- ---------- ---------- ---------- --------MA VKKS------ ----VGSLKE
XP_0219934  ---------- ---------- ---------- ---------- ---------- ---------- --------MA TKKS------ ----VSSLKE
XP_0220396  ---------- ---------- ---------- ---------- ---------- ---------- --------MA TKKS------ ----VSSLTE
at_cPGK2    FGHIP--STS VSARRLGFSA --VVDS-RFS VHVASKVHSV R---G----- ---------- -KGARGVITM AKKS------ ----VGDLNS
Alyrata_cP  FLPIP--STA VSARRLGFSA --FVDS-RFS VHVASKVQSV R---G----- ---------- -KGTRGVITM AKKS------ ----VGDLTS
At_cPGK1    LLPIP--STS VSARPLGFSA --TLDSRRFS LHVASKVESV R---G----- ---------- -KGSRGVVSM AKKS------ ----VGDLTS
Alyr_cPGK1  LLPVP--STS VSARPLGFSA --TLDSRRFT LHVASKVESV R---G----- ---------- -KGSRGVVSM AKKS------ ----VGDLTS
XP_0136482  LLPITPSSKS ISPRPLGFSA --VLDSHRFS LHVASKVHSV R---G----- ---------- -RGTRGVVSM AKKI------ ----VGDLTS
XP_0091352  LLPITPSSKS ISPRPLGFSA --VLDSHRFS LHVASKVHSV R---G----- ---------- -RGSRGVVSM AKKS------ ----VGDLTS
XP_0225670  LLPVP--TKS ISTRPLGFSA --VLDS-RFT VHVASKVQSF R---G----- ---------- -KGTRGVVSM AKKS------ ----VGDLTS
XP_0136731  LLPVP--SKS ISTRPLGFSA --VLDS-RFT VHVASKVQSF R---G----- ---------- -KGTRGVVSM AKKS------ ----VGDLTS
XP_0091246  LLPIP--SKS ISTRPLGFSA --VLDS-RFT VHVASKVQSF R---G----- ---------- -KGTRGVVSM AKKS------ ----VGDLTS
XP_0136856  LLPITSKSKS ISTRPLGFSA --VLDS-RFT VHVSSKVQSF R---G----- ---------- -KGTRGVVSM A-KS------ ----VGDLTS
XP_0091465  LLPISSKSKS ISTRPLGFSA --VLDS-RFT VHVASKVQSF R---G----- ---------- -KGTRGVVSM AKKS------ ----VGDLTS
XP_0084563  TSSSSIRLRS AATPRLGFAA ----ADPLFS LHVASKIRSF G---G----- ---------- -KASRGVVSM AKKS------ ----VGDLTA
XP_0165815  LLKTP----- --LRRLGFAG --AVADSLFT SHVATRLRSF SGSSS----- ---------- -KPVRAVVSM AKKS------ ----VGDLTA
PHT77520.1  LLKTP----- --LRRLGFAG --AVADSLFT SHVATRLRSF SGSSS----- ---------- -KPVRAVVSM AKKS------ ----VGDLTA
XP_0042439  L--------K TPLRRLGFAG --TVADPLFT NHVATKLRSV K-ASS----- ---------- -NPVRAVVSM AKKS------ ----VGDLSS
NP_0012752  LLKTP----- --LRRLGFAG --AVADPLFT NHVATKLRSL K-ASS----- ---------- -NPVRAVVSM AKKS------ ----VGDLSS
XP_0151700  LLKTP----- --LRRLGFAG --AVADPLFT NHVATKLRSL K-ASS----- ---------- -NPVRAVVSM AKKS------ ----VGDLSS
ADR71054.1  FLAKT----- -PPRGLGFAG --AAADSLFT NHVATKLRSL K-SSS----- ---------- -KPVRGVASM AKKS------ ----VGDLTA
XP_0164941  FLAKT----- -PLRRLGFAG --AAADSLFT NHVATKLRSL KSSS------ ---------- -KPIRGVASM AKKS------ ----VGDLTA
XP_0220015  FLRSP----- --LRGLGFAA ----ADTAFS AHVASKLRSC STSV------ ---------- -KPISRGVFA AKKS------ ----VGDLSP
AQK86959.1  LRRGGLATAC QPARSLAFAA ----GDARLA VHVASRCRQA F--SG----- ---------- -RGTRAMATM AKKS------ ----VGELTE
Zm00001d01  ---------- ---------- ---------- ---------- ---------- ---------- ---------- ---------- ----------
A8JC04_cPG  PRVVPFSSAS SSVLRSGFAA ---------- -EVSVDIRRV G--------- ---------- --RSRIVVEA VKKS------ ----VGDLHK
GAQ79801.1  ---------- ---------- ---------- ---------- ---------- ---------- ---------- ---------- ----LNDVDK
sp_P18912.  ---------- ---------- ---------- ---------- ---------- ---------- ---------M NKKT------ -------IRD
AAC75963.1  ---------- ---------- ---------- ---------- ---------- ---------- ---------- --MS------ ----VIKMTD
sp|P50315|  ---------- ---------- ---------- ---------- ---------- ---------- --------MM TFQT------ ----LDDLDD
sp_P00560.  ---------- ---------- ---------- ---------- ---------- ---------- --------SL SSKL------ ------SVQD
NP_112467.  ---------- ---------- ---------- ---------- ---------- ---------- --------AL SAKL------ ------TLDK
sp|P07205.  ---------- ---------- ---------- ---------- ---------- ---------- --------SL SKKL------ ------TLDK
sp|P00558.  ---------- ---------- ---------- ---------- ---------- ---------- --------SL SNKL------ ------TLDK
NP_032854.  ---------- ---------- ---------- ---------- ---------- ---------- -------MSL SNKL------ ------TLDK
Zm00001d03  ---------- ---------- ---------- ---------- ---------- ---------- ---------- ---------- ----------
tr_A0A1S4D  YDRYHTVLMS TPARSWGAVY CKNLVNSEDH ASSWWNLEAV P--------- ---------- ---------- HVQT------ ----LGEFPR
XP_0165427  QIRLCLSYGK CSVVQKCKKA QFLLKALNLD LADHVDLHSF REECAVRSSY PNKNPLLAEA CDGRDSDILP HVQT------ ----LKNFPA
XP_0063543  ---------- ---------- ---------- ---------- ---------- ---------- ---------- ---------- ----------
XP_0258888  YGKCSAVRKC KKIRFLRQAL NLELIDHADL LSFPNEAEAC D---G----- ---------- -RGTDILPYV QT-------- ----VRNFPE
XP_0042466  YGKCSAVRKC KKIRFLRQAL NLELIDHADL LSFPNEAEAC ---------- ---------- -DGRGTDILP YVQT------ ----VRNFPE
KAF3623713  LLKTP----- --LRRLGFAG --AVADSLFT SHVATRLRSF SGSSS----- ---------- -KPVRAVVSM AKKS------ ----VGDLTA
GAQ85336.1  RKPCFRLVGT CQLTANGQGA SEQVETRGLE QEAQKYESPS F--------- ---------- -LPSRQQLNM PPRDERVSYT RIQGLSTFPP
A8JFT4_PGK  ---------- ---------- ---------- ---------- ---------- ---------- -RLLRDVVND LIAP------ ----ELDVAN
```

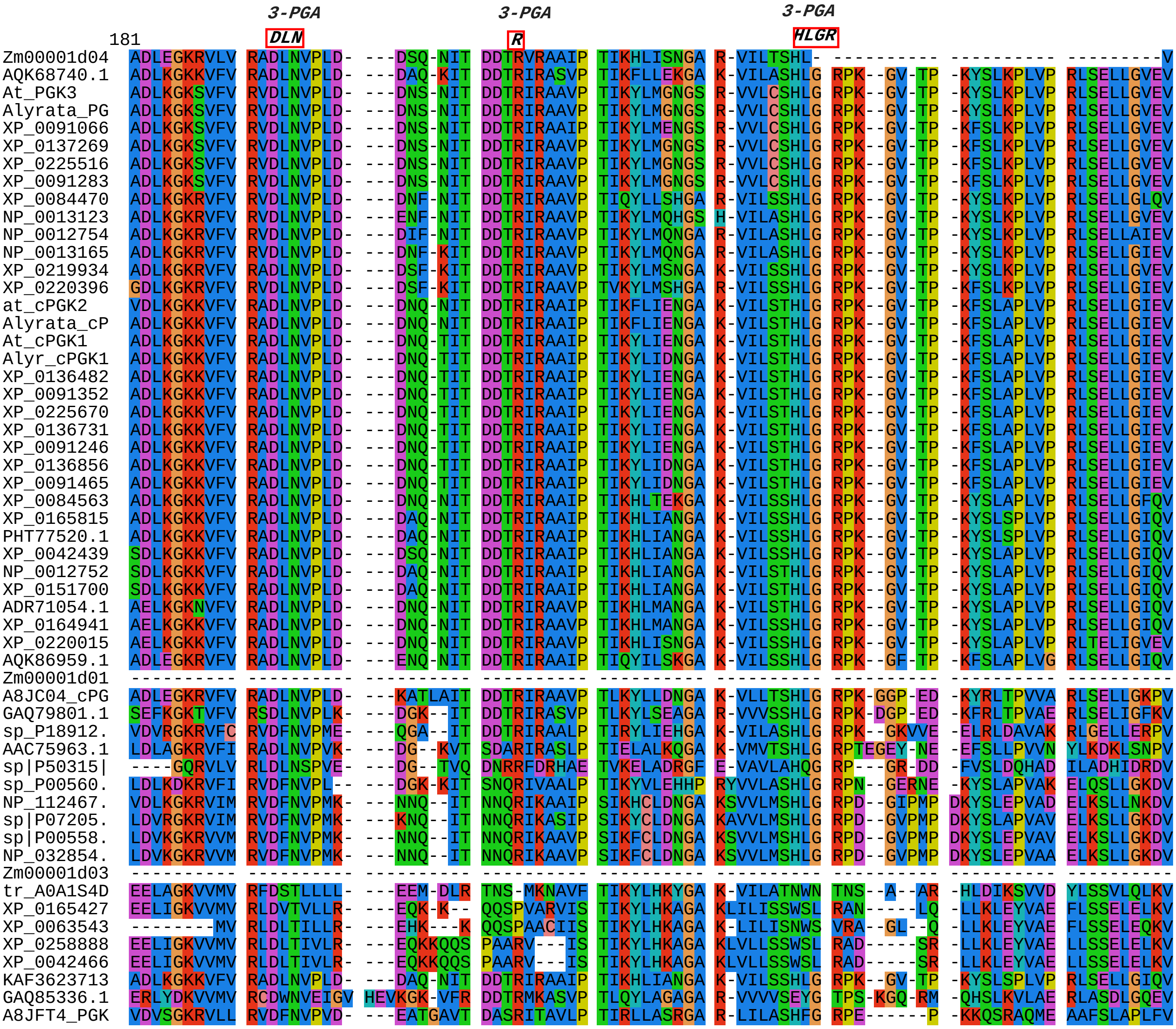
3-PGA
3-PGA
3-PGA
181
DLN
R
HLGR
Zm00001d04
AQK68740.1
At_PGK3
Alyrata_PG
XP_0091066
XP_0137269
XP_0225516
XP_0091283
XP_0084470
NP_0013123
NP_0012754
NP_0013165
XP_0219934
XP_0220396
at_cPGK2
Alyrata_cP
At_cPGK1
Alyr_cPGK1
XP_0136482
XP_0091352
XP_0225670
XP_0136731
XP_0091246
XP_0136856
XP_0091465
XP_0084563
XP_0165815
PHT77520.1
XP_0042439
NP_0012752
XP_0151700
ADR71054.1
XP_0164941
XP_0220015
AQK86959.1
Zm00001d01
A8JC04_cPG
GAQ79801.1
sp_P18912.
AAC75963.1
sp|P50315|
sp_P00560.
NP_112467.
sp|P07205.
sp|P00558.
NP_032854.
Zm00001d03
tr_A0A1S4D
XP_0165427
XP_0063543
XP_0258888
XP_0042466
KAF3623713
GAQ85336.1
A8JFT4_PGK

```
                                                     3-PGA                                                    3-PGA
             271                                       R                                                          R
Zm00001d04   IKADDVVGPE VEKLVSALPN GNVVLLENVR FYKEEE---- -------KND PE----FAKK LASLADLY-- ---------- ----------
AQK68740.1   VMANDCIGEE VEKLAAALPE GGVLLLENVR FYKEEE---- -------KNE PE----FAKK LASVADLYVN DAFGTAHRAH ASTEGVTKYL
At_PGK3      VMANDSIGEE VQKLVAGLPE GGVLLLENVR FYAEEE---- -------KND PE----FAKK LAALADVYVN DAFGTAHRAH ASTEGVAKFL
Alyrata_PG   VMANDSIGEE VQKLVAGLPE GGVLLLENVR FYAEEE---- -------KND PE----FAKK LAALADVYVN DAFGTAHRAH ASTEGVAKYL
XP_0091066   VMANDSIGEE VQKLVAGLPE GGVLLLENVR FYKEEE---- -------KND PE----FAKK LAALADVYVN DAFGTAHRAH ASTEGVAKYL
XP_0137269   VMADDSIGEE VQKLVAGLPE GGVLLLENVR FYKEEE---- -------KND PE----FAKK LAALADVYVN DAFGTAHRAH ASTEGVAKYL
XP_0225516   VMANDSIGEE VQKLVAGLPE GGVLLLENVR FYKEEE---- -------KND PE----FAKK LAAPADVYVN DAFGTAHRAH ASTEGVAKYL
XP_0091283   VMANDSIGEE VQKLVAGLPE GGVLLLENVR FYKEEE---- -------KND PE----FAKK LAALADVYVN DAFGTAHRAH ASTEGVAKYL
XP_0084470   IMANDCIGEE VEKMVAELPE GGVLLLENVR FYKEEE---- -------KND PE----FAKK LASLADLYVN DAFGTAHRAH ASTEGVAKYL
NP_0013123   KIANDSIGPE VEKLVAEIPE GGVLLLENVR FYKEEE---- -------KNE PE----FAKK LASLADLYVN DAFGTAHRAH ASTEGVAKYL
NP_0012754   KMANDSIGPE VENLVAELPE GGVVLLENVR FYKEEE---- -------KND PE----FAKK LASLADLYVN DAFGTAHRAH ASTEGVAKVL
NP_0013165   KMANDSVGPE VENLVAGLPE GGVVLLENVR FYKEEE---- -------KND PE----FAKK LASLADLYVN DAFGTAHRAH ASTEGVAKVL
XP_0219934   KMADDCVGPE VEKLVAGIPE GGVLLLENVR FYKEEE---- -------KND PE----FAKK LASLADLYVN DAFGTAHRAH ASTEGVAKYL
XP_0220396   KMADDCIGPD VEKLVAEIPE GGVLLLENVR FYKEEE---- -------KND PE----FAKK LASLADLYVN DAFGTAHRAH ASTEGVAKHL
at_cPGK2     VKADDCIGPE VETLVASLPE GGVLLLENVR FYKEEE---- -------KNE PD----FAKK LASLADLYVN DAFGTAHRAH ASTEGVTKFL
Alyrata_cP   VKADDCIGPQ VESLVASLPE GGVLLLENVR FYKEEE---- -------KNE PE----FAKK LASLADLYVN DAFGTAHRAH ASTEGVTKFL
At_cPGK1     TKADDCIGPE VESLVASLPE GGVLLLENVR FYKEEE---- -------KND PE----FAKK LASLADLYVN DAFGTAHRAH ASTEGVTKFL
Alyr_cPGK1   TKADDCIGPE VESLVASLPE GGVLLLENVR FYKEEE---- -------KND PE----FAKK LASLADLYVN DAFGTAHRAH ASTEGVTKFL
XP_0136482   KKADDCIGPE VESLVASLPE GGVLLLENVR FYKEEE---- -------KND PE----FAKK LASLADLYVN DAFGTAHRAH ASTEGVTKFL
XP_0091352   KKADDCIGPE VESLVASLPE GGVLLLENVR FYKEEE---- -------KND PE----FAKK LASLADLYVN DAFGTAHRAH ASTEGVTKFL
XP_0225670   KKADDCIGPE VESLVASLPE GGVLLLENVR FYKEEE---- -------KND PD----FAKK LASLADLYVN DAFGTAHRAH ASTEGVTKFL
XP_0136731   KKADDCIGPE VESLVASLPE GGVLLLENVR FYKEEE---- -------KND PE----FAKK LASLADLYVN DAFGTAHRAH ASTEGVTKFL
XP_0091246   KKADDCIGPE VESLVASLPE GGVLLLENVR FYKEEE---- -------KND PE----FAKK LASLADLYVN DAFGTAHRAH ASTEGVTKFL
XP_0136856   KKADDCIGPE VESLVGSLPE GGVLLLENVR FYQEEE---- -------KND PE----FAKK LASLADLYVN DAFGTAHRAH ASTEGVTKFL
XP_0091465   KKADDCIGPE VESLVGSLPE GGVLLLENVR FYKEEE---- -------KND PE----FAKK LASLADLYVN DAFGTAHRAH ASTEGVTKFL
XP_0084563   VKADDCIGPE VEKLVASLPE GGVLLLENVR FYKEEE---- -------KNE PE----FAKK LASLADLFVN DAFGTAHRAH ASTEGVTKFL
XP_0165815   VKADDCIGPE VEKLVASLPE GGVLLLENVR FYKEEE---- -------KNE PE----FAKK LAALADLYVN DAFGTAHRAH ASTEGVTKFL
PHT77520.1   VKADDCIGPE VEKLVASLPE GGVLLLENVR FYKEEE---- -------KNE PE----FAKK LAALADLYVN DAFGTAHRAH ASTEGVTKFL
XP_0042439   VKADDCIGPE VDKLVDSLPE GGVLLLENVR FYKEEE---- -------KNE PE----FAKK LASLADLYVN DAFGTAHRAH ASTEGVTKFL
NP_0012752   VKADDCIGPE VEKLVDSLPE GGVLLLENVR FYKEEE---- -------KNE PE----FXKK LASLADLYVN DAFGTAHRAH ASTEGVTKFL
XP_0151700   VKADDCIGPE VEKLVDSLPE GGVLLLENVR FYKEEE---- -------KNE PE----FAKK LASLADLFVN DAFGTAHRAH ASTEGVTKFL
ADR71054.1   VKVEDCIGPE VEKLVASLPE GGVLLLENVR FYKEEE---- -------KNE PE----FAKK LASLADLYVN DAFGTAHRAH ASTEGVTKFL
XP_0164941   VKAEDCIGPE VEKLVASLPE GGVLLLENVR FYKEEE---- -------KNE PE----FAKK LASLADLYVN DAFGTAHRAH ASTEGVTKFL
XP_0220015   VKADDCIGPD VEKLVASLPD GGVLLLENVR FYKEEE---- -------KND PG----FAEK LASLADLYVN DAFGTAHRAH ASTEGVTKFL
AQK86959.1   QKADDVIGPE VEKLVAALPN GGVLLLENVR FYKEEE---- -------KND PE----FAQK LASLADLYVN DAFGTAHRAH ASTEGVTKFL
Zm00001d01   ---------- ---------- ---------- ---------- ---------- ---------- ---------- ---------- ----------
A8JC04_cPG   TKVDDCIGPE VEKAVGAMKN GELLLLENVR FYKEEE---- -------KNE PE----FAKK LAANADLYVN DAFGTAHRAH ASTEGVTKFL
GAQ79801.1   EKANDSIGDE VQKQIKDLKD GGVLLLENVR FYKEEE---- -------KND PE----HAKA LGKGIDYFVN DAFGTAHRAH ASTAGIANYV
sp_P18912.   AKTNEAVGDE VKAAVDRLNE GDVLLLENVR FYPGEE---- -------KND PE----LAKA FAELADLYVN DAFGAAHRAH ASTEGIAHYL
AAC75963.1   RLVKDYLDG- -----VDVAE GELVVLENVR FNKGEK---- -------KDD ET----LSKK YAALCDVFVM DAFGTAHRAQ ASTHGIGKFA
sp|P50315|   DFVDETYGPQ AIHDIADLDS GDVLVLENTR MCDDEL---- PEEDPEVKAQ TE----FVKT LAGEFDAYIN DAYSAAHRSH ASLVGFP-LV
sp_P00560.   TFLNDCVGPE VEAAVKASAP GSVILLENLR YHIEEE-GSR KVDGQKVKAS KEDVQKFRHE LSSLADVYIN DAFGTAHRAH SSMVGFD--L
NP_112467.   IFLKDCVGPE VEQACANPDN GSIILLENLR FHVEEEGKGK DSSGKKISAD PAKVEAFQAS LSKLGDVYVN DAFGTAHRAH SSTVGVN--L
sp|P07205.   LFLKDCVGAE VEKACANPAP GSVILLENLR FHVEEEGKGQ DPSGKKIKAE PDKIEAFRAS LSKLGDVYVN DAFGTAHRAH SSMVGVN--L
sp|P00558.   LFLKDCVGPE VEKACANPAA GSVILLENLR FHVEEEGKGK DASGNKVKAE PAKIEAFRAS LSKLGDVYVN DAFGTAHRAH SSMVGVN--L
NP_032854.   LFLKDCVGPE VENACANPAA GTVILLENLR FHVEEEGKGK DASGNKVKAE PAKIDAFRAS LSKLGDVYVN DAFGTAHRAH SSMVGVN--L
Zm00001d03   ---------- ---------- ---------- ---------- ---------- ---------- -----MIFVN DSFSLSHKIL ASTVGITCFC
tr_A0A1S4D   CPVK-CSSYS MMLTEEGFEK ADILLLENLS MFKEEV---- -------ANC SK----FSKM LSLGVDIFVN DSFSQSHRIL ASTVGITRFC
XP_0165427   IPVELGSGLE -QYVLEDRHK SDILLLENLS QFKQEQ---- -------ANC SE----FARR LSSGVDIFVN DAFFQSHKIL ASNVGITGFC
XP_0063543   IPVEFGSGFE -QSLMEDRHK SDILLLENLS HFKQEQ---- -------ANS SE----FSRR LSSGVDIFVN DTFFQSHKIL ASNVGITGFC
XP_0258888   IPVDLGSGFE -QSLREDRHK SSILLLENLS QFKQEQ---- -------ANC SE----FARR LSSGVDIFVN ETFFQSHKIL ASNVGITGFC
XP_0042466   IPVDLGSGFE -QSLREDRHK SSILLLENLS QFKQEQ---- -------ANC SE----FARR LSSGVDIFVN ETFFQSHKIL ASNVGITGFC
KAF3623713   VKADDCIGPE VEKLVASLPE GGVLLLENVR FYKEEE---- -------KNE PE----FAKK LAALADLYVN DAFGTAHRAH ASTEGVTKFL
GAQ85336.1   MMAGDCIGAG VERDIDALRP GQILVLENVR FYKEEK---- -------AND LV----FAQK LAFRVDVFVN DALGPCSQAT ASVSGVPVFV
A8JFT4_PGK   GLAGDCIGPE AEAAVAALQP GQVLLLQNTR FHAGDG---- -------AND SG----FSGA LAALCDVFVQ DAFGVVHRDQ GSVTGITAHV
```

```
                                                                                                            ATP
                 361                                                                                         G
Zm00001d04  ---------- QELDYLVGAV SNP-KRPFAA VI-------- ---------- --GGSKVSSK IGVIESLL-- EKCDILLLGG GMIFTFYKA-
AQK68740.1  KPAVAGFLMQ KELDYLVGAV ANP-KKPFAA IV-------- ---------- --GGSKVSTK IGVIESLL-- AKVDILILGG GMIYTFYKA-
At_PGK3     KPSVAGFLMQ KELDYLVGAV ANP-KKPFAA IV-------- ---------- --GGSKVSTK IGVIESLL-- NTVDILLLGG GMIFTFYKA-
Alyrata_PG  KPSVAGFLMQ KELDYLVGAV ANP-KKPFAA IV-------- ---------- --GGSKVSTK IGVIESLL-- STVDILLLGG GMIFTFYKA-
XP_0091066  KPSVAGFLMQ KELDYLVGAV ANP-KKPFAA IV-------- ---------- --GGSKVSTK IGVIESLL-- STVDILLLGG GMIFTFYKA-
XP_0137269  KPSVAGFLMQ KELDYLVGAV ANP-KKPFAA IV-------- ---------- --GGSKVSTK IGVIESLL-- STVDILLLGG GMIFTFYKA-
XP_0225516  KPSVAGFLMQ KELDYLVGAV ANP-KKPFAA IV-------- ---------- --GGSKVSTK IGVIESLL-- STVDILLLGG GMIFTFYKA-
XP_0091283  KPSVAGFLMQ KELDYLVGAV ANP-KKPFAA IV-------- ---------- --GGSKVSTK IGVIESLL-- STVDILLLGG GMIFTFYKA-
XP_0084470  KPSVAGFLMQ KELDYLVGAV SNP-KRPFAA IV-------- ---------- --GGSKVSSK IGVIESLL-- EKVNLLFLGG GMIFTFYKA-
NP_0013123  KPAVAGFLMQ KELDYLVGAV ANP-QKPFAA IV-------- ---------- --GGSKVSSK IGVIESLL-- EKVDVLLLGG GMIFTFYKA-
NP_0012754  KPAVAGFLMQ KELDYLVGAV SNP-QKPFAA IV-------- ---------- --GGSKVSSK IGVIESLL-- EKVDVLLLGG GMIFTFYKA-
NP_0013165  KPAVAGFLMQ KELDYLVGAV SNP-QKPFAA IV-------- ---------- --GGSKVSSK IGVIESLL-- EKVDVLLLGG GMIFTFYKA-
XP_0219934  KPSVAGYLMQ KELDYLVGAV SNP-TKPFAA IV-------- ---------- --GGSKVSSK IGVIESLL-- EKVNILVLGG GMIFTFYKA-
XP_0220396  KPAVAGFLMQ KELDYLVGAV SNP-KKPFAA IV-------- ---------- --GGSKVSSK IGVIESLL-- EKVDILVLGG GMIFTFYKA-
at_cPGK2    KPSVAGFLLQ KELDYLVGAV SNP-KRPFAA IV-------- ---------- --GGSKVSSK IGVIESLL-- EKCDILLLGG GMIFTFYKA-
Alyrata_cP  KPSVAGFLLQ KELDYLVGAV SNP-KRPFAA IV-------- ---------- --GGSKVSSK IGVIESLL-- EKCDILLLGG GMIFTFYKA-
At_cPGK1    KPSVAGFLLQ KELDYLVGAV SNP-KRPFAA IV-------- ---------- --GGSKVSSK IGVIESLL-- EKCDILLLGG GMIFTFYKA-
Alyr_cPGK1  KPSVAGFLLQ KELDYLVGAV SNP-KRPFAA IV-------- ---------- --GGSKVSSK IGVIESLL-- EKCDILLLGG GMIFTFYKA-
XP_0136482  KPSVAGFLLQ KELDYLVGAV SNP-KRPFAA IV-------- ---------- --GGSKVSSK IGVIESLL-- EKCDILLLGG GMIFTFYKA-
XP_0091352  KPSVAGFLLQ KELDYLVGAV SNP-KRPFAA IV-------- ---------- --GGSKVSSK IGVIESLL-- EKCDILLLGG GMIFTFYKA-
XP_0225670  KPSVAGFLLQ KELDYLVGAV SSP-KRPFAA IV-------- ---------- --GGSKVSSK IGVIESLL-- EKCDILLLGG GMIFTFYKA-
XP_0136731  KPSVAGFLLQ KELDYLVGAV SSP-KRPFAA IV-------- ---------- --GGSKVSSK IGVIESLL-- EKCDILLLGG GMIFTFYKA-
XP_0091246  KPSVAGFLLQ KELDYLVGAV SSP-KRPFAA IV-------- ---------- --GGSKVSSK IGVIESLL-- EKCDILLLGG GMIFTFYKA-
XP_0136856  KPSVAGFLLQ KELDYLVGAV SSP-KRPFAA IV-------- ---------- --GGSKVSSK IGVIESLL-- EKCDILLLGG GMIFTFYKA-
XP_0091465  KPSVAGFLLQ KELDYLVGAV SSP-KRPFAA IV-------- ---------- --GGSKVSSK IGVIESLL-- EKCDILLLGG GMIFTFYKA-
XP_0084563  KPSVAGFLLQ KELDYLVGAV SSP-KRPFAA IV-------- ---------- --GGSKVSSK IGVIESLL-- EKCDILLLGG GMIFTFYKA-
XP_0165815  KPSVAGFLLQ KELDYLVGAV STP-KRPFAA IV-------- ---------- --GGSKVSSK IGVIESLL-- EKCDILLLGG GMIFTFYKA-
PHT77520.1  KPSVAGFLLQ KELDYLVGAV STP-KRPFAA IV-------- ---------- --GGSKVSSK IGVIESLL-- EKCDILLLGG GMIFTFYKA-
XP_0042439  KPSVAGFLLQ KELDYLVGAV STP-KRPFAA IV-------- ---------- --GGSKVSSK IGVIESLL-- EKCDILLLGG GMIFTFYKA-
NP_0012752  KPSVAGFLLQ KELDYLVGAV STP-KRPFAA IV-------- ---------- --GGSKVSSK IGVIESLL-- EKCDILLLGG GMIFTFYKA-
XP_0151700  KPSVAGFLLQ KELDYLVGAV STP-KRPFAA IV-------- ---------- --GGSKVSSK IGVIESLL-- EKCDILLLGG GMIFTFYKA-
ADR71054.1  KPSVAGFLLQ KELDYLVGAV SNP-KRPFAA IV-------- ---------- --GGSKVSSK IGVIESLL-- EKCDILLLGG GMIFTFYKA-
XP_0164941  KPSVAGFLLQ KELDYLVGAV SNP-KRPFAA IV-------- ---------- --GGSKVSSK IGVIESLL-- EKCDILLLGG GMIFTFYKA-
XP_0220015  KPSVAGFLLQ KELDYLDGAV SNP-KRPFAA IV-------- ---------- --GGSKVSSK IGVIESLL-- EKCDILLLGG GMIFTFYKA-
AQK86959.1  QPSVAGFLLQ KELDYLVGAV SSP-KRPFAA IV-------- ---------- --GGSKVSSK IGVIESLL-- EKCDILLLGG GMIFTFYKA-
Zm00001d01  ---------- ---------- ---------- ---------- ---------- ---------- ---------- ---------- -MIFTFYKA-
A8JC04_cPG  KPSVAGFLLQ KELDYLDGAV SNP-KRPFVA IV-------- ---------- --GGSKVSSK ITVIEALM-- EKCDKIIIGG GMIFTFYKA-
GAQ79801.1  TRSIAGFLLE KELAYLEGAV KAP-KRPFVA IV-------- ---------- --GGSKVSSK IGVIESLI-- GSVDKIILGG GMIFTFYKS-
sp_P18912.  -PAVAGFLME KELEVLGKAL SNP-DRPFTA II-------- ---------- --GGAKVKDK IGVIDNLL-- EKVDNLIIGG GLAYTFVKA-
AAC75963.1  DVACAGPLLA AELDALGKAL KEP-ARPMVA IV-------- ---------- --GGSKVSTK LTVLDSLS-- KIADQLIVGG GIANTFIAA-
sp|P50315|  MDAYAGRVME TEYEANTAIA EKEFDGQVTM VV-------- ---------- --GGTKATDV IDVMTHLD-- EKVDDFLLGG -IAGTVPAA-
sp_P00560.  PQRAAGFLLE KELKYFGKAL ENP-TRPFLA IL-------- ---------- --GGAKVADK IQLIDNLL-- DKVDSIIIGG GMAFTFKKVL
NP_112467.  PQKASGFLMK KELDYFSKAL EKP-ERPFLA IL-------- ---------- --GGAKVKDK IQLIKNML-- DKVNFMIIGG GMAYTFLKEL
sp|P07205.  PHKASGFLMK KELDYFAKAL ENP-VRPFLA IL-------- ---------- --GGAKVADK IQLIKNML-- DKVNEMIIGG GMAYTFLKVL
sp|P00558.  PQKAGGFLMK KELNYFAKAL ESP-ERPFLA IL-------- ---------- --GGAKVADK IQLINNML-- DKVNEMIIGG GMAFTFLKVL
NP_032854.  PQKAGGFLMK KELNYFAKAL ESP-ERPFLA IL-------- ---------- --GGAKVADK IQLINNML-- DKVNEMIIGG GMAFTFLKVL
Zm00001d03  YASLAGFHFE EELMKLIKIT DST-RRPYIA IVIINLFFPD LYFQYSINVC KIGGSNFLRK APALQTLT-- CLCDGLFFVG KLSFQIMNG-
tr_A0A1S4D  STCLAGFNFQ ERLCQLKKAA ETK-RQPYVA II-------- ---------- --GGGNLLDK LAALHVLA-- STCSALVFVG MMSFQIMQA-
XP_0165427  YASIAGFHFE EGMSQLKKII EMN-KRPYFA IV-------- ---------- --GGANLAGK AAALQFLA-- SRCDGLVFVG DAAFQIMHA-
XP_0063543  YASIAGFHFD EQMSQLKKII EMN-KRPYFA IV-------- ---------- --GGN-LAGK TAALLFLA-- SRCDGLVFVG DAAFQIMHA-
XP_0258888  YASIAGFHFD EQMSQLKKII EMN-KRPYFA IV-------- ---------- --GGNLAGKA VALL-FLA-- SRCDGLVFIG NAAFQIMHA-
XP_0042466  YASIAGFHFD EQMSQLKKII EMN-KRPYFA IV-------- ---------- --GGNLAGKA VALL-FLA-- SRCDGLVFIG NAAFQIMHA-
KAF3623713  KPSVAGFLLQ KELDYLVGAV STP-KRPFAA IV-------- ---------- --GGSKVSSK IGVIESLL-- EKCDILLLGG GMIFTFYKA-
GAQ85336.1  GRRIAGLQLE KELRYLNSLT SSA-ARPLLA IV-------- ---------- --GGGKLSTR FPLLKSLIAH QKCDAILLGS ALSAPFLRA-
A8JFT4_PGK  SECYPGPLVR RELTELAHRL FEP-VRPLGV VL-------- ---------- --GGAKVADK IGVVAALV-- EMADVVAVGG RMAFTLLAA-
```

```
                                                                                                     ATP
                                                                                                      G
           451
Zm00001d04 QGHSIG---- -SSLVEDDKL ELATSLLRKA KEKGVSLMLP FDLVVADKFA PDASS----- ---------- ---------- ----------
AQK68740.1 QGYSVG---- -KSLVEEDKL ELATSLIEKA KAKGVSLLLP TDIVVADKFA ADAESKI--- -----VP-AT AIPDDWMG-L DVGPDATKTF
At_PGK3    QGLSVG---- -SSLVEEDKL DLAKSLMEKA KAKGVSLLLP TDVVIADKFA PDANSKI--- -----VP-AT AIPDGWMG-L DIGPDSIKTF
Alyrata_PG QGLSVG---- -SSLVEEDKL DLAKSLMEKA KAKGVSLLLP TDVVIADKFA PDANSKI--- -----VP-AT AIPDGWMG-L DIGPDSIKTF
XP_0091066 QGHSVG---- -SSLVEEDKL DLAKSLMEKA KAKGVSLLLP TDVVIADKFA PDANSKI--- -----VA-AT AIPDGWMG-L DIGPDSIKTF
XP_0137269 QGHSVG---- -SSLVEEDKL DLAKSLMEKA KAKGVSLLLP TDVVIADKFA PDANSKI--- -----VA-AT AIPDGWMG-L DIGPDSIKTF
XP_0225516 QGHSVG---- -SSLVEEDKL DLAKSLMEKA KAKGVSLLLP TDVVIADKFA PDANSKI--- -----VP-AT AIPDGWMG-L DVGPDSIKTF
XP_0091283 QGHSVG---- -SSLVEEDKL DLAKSLMEKA KAKGVSLMLP TDVVIADKFA PDANSKI--- -----VP-AT AIPDGWMG-L DVGPDSIKTF
XP_0084470 QGLAVG---- -KSLVEEDKL DLATSLIEKA KAKGVSLLLP TDVVVADKFA ADADSKV--- -----VA-AS SIPDGWMG-L DIGPDSIKSF
NP_0013123 QGYAVG---- -SSLVEEDKL DLATSLMEKA KAKGVSLLLP TDVVIADKFA ADANSKV--- -----VP-AS EIPDGWMG-L DIGPDAIKSF
NP_0012754 QGYAVG---- -SSLVEEDKL DLATSLMEKA KTKGVSLLLP TDVVIADKFA ADANSKI--- -----VP-AS EIPDGWMG-L DIGPDAIKSF
NP_0013165 QGYAVG---- -SSLVEEDKL DLATSLMEKA KTKGVSLLLP TDVVIADKFA ADANSKI--- -----VP-AS EIPDGWMG-L DIGPDAIKSF
XP_0219934 QGYKVG---- -SSLVEEDKL DLATSLIEKA KAKGVSLLLP SDVVIADKFA ADANSKV--- -----VP-AK EIPDGWMG-L DIGPDSIKSF
XP_0220396 QGLAVG---- -SSLVEEDKL DLATTLLEKA KSKGVSLLLP SDVVIADKFA ADANSKI--- -----VP-AS SIPDGWMG-L DIGPDSIKSF
at_cPGK2   QGLSVG---- -SSLVEEDKL ELATTLLAKA KARGVSLLLP TDVVIADKFA PDANSKI--- -----VP-AS AIPDGWMG-L DIGPDSVKTF
Alyrata_cP QGLSVG---- -SSLVEEDKL ELATSLLAKA KARGVSLLLP TDVVIADKFA PDANSKI--- -----VP-AS AIPDGWMG-L DIGPDSVKTF
At_cPGK1   QGLSVG---- -SSLVEEDKL ELATELLAKA KAKGVSLLLP TDVVIADKFA PDANSKI--- -----VP-AS GIEDGWMG-L DIGPDSIKTF
Alyr_cPGK1 QGLSVG---- -SSLVEEDKL ELATELLAKA KAKGVSLLLP TDVVVADKFA PDANSKI--- -----VP-AS GIEDGWMG-L DIGPDSIKTF
XP_0136482 QGLSVG---- -SSLVEEDKL ELATSLLAKA KAKGVSLLLP TDVVVADKFA PDANSKV--- -----VP-AS GIEDGWMG-L DIGPDSIKTF
XP_0091352 QGLSVG---- -SSLVEEDKL ELATSLLAKA KAKGVSLLLP TDVVVADKFA PDANSKV--- -----VP-AS GIEDGWMG-L DIGPDSIKTF
XP_0225670 QGLSVG---- -SSLVEEDKL ELATSLLAKA KAKGVSLLLP TDVVVADKFA PDANSKV--- -----VS-AS GIEDGWMG-L DIGPDSIKTF
XP_0136731 QGLSVG---- -SSLVEEDKL ELATSLLAKA KAKGVSLLLP TDVVVADKFA PDANSKV--- -----VS-AS GIEDGWMG-L DIGPDSIKTF
XP_0091246 QGLSVG---- -SSLVEEDKL ELATSLLAKA KAKGVSLLLP TDVVVADKFA PDANSKV--- -----VS-AS GIEDGWMG-L DIGPDSIKTF
XP_0136856 QGLSVG---- -SSLVEEDKL ELATSLLAKA KAKGVSLLLP TDVVVADKFA PDANSKV--- -----VS-AS GIEDGWMG-L DIGPDSIKTF
XP_0091465 QGLSVG---- -SSLVEEDKL ELATSLLAKA KAKGVSLLLP TDVVVADKFA PDANSKV--- -----VS-AS GIEDGWMG-L DIGPDSIKTF
XP_0084563 QGLSVG---- -SSLVEEDKL ELATSLLEKA KAKGVSLLLP TDVIIADKFA PDANSKI--- -----VP-AS AIPDGWMG-L DIGPDSVKTF
XP_0165815 QGLSVG---- -SSLVEEDKL DLATSLLEKA KAKGVSLLLP SDVVIADKFA PDANSKI--- -----VP-AS AIPDGWMG-L DIGPDSVKTF
PHT77520.1 QGLSVG---- -SSLVEEDKL DLATSLLEKA KAKGVSLLLP SDVVIADKFA PDANSKI--- -----VP-AS AIPDGWMG-L DIGPDSVKTF
XP_0042439 QGLSVG---- -SSLVEEDKL ELATSLLEKA KAKGVSLLLP SDVVIADKFA PDANSKI--- -----VP-AS AIPDGWMG-L DIGPDSIKTF
NP_0012752 QGLSVG---- -SSLVEEDKL ELATSLLEKA KAKGVSLLLP SDVVIADKFA PDANSKI--- -----VP-AS AIPDGWMG-L DIGPDSIKTF
XP_0151700 QGLSVG---- -SSLVEEDKL ELATSLLEKA KAKGVSLLLP SDVVIADKFA PDANSKI--- -----VP-AS AIPDGWMG-L DIGPDSIKTF
ADR71054.1 QGLSVG---- -SSLVEEDKL ELATSLLEKA KAKGVSLLLP SDVVIADKFA PDANSKI--- -----VP-AS AIPDGWMG-L DIGPDSVKTF
XP_0164941 QGLSVG---- -SSLVEEDKL ELATSLLEKA KAKGVSLLLP SDVVIADKFA PDANSKI--- -----VP-AS AIPDGWMG-L DIGPDSVKTF
XP_0220015 QGLSVG---- -SSLVEEDKL DLATTLLAKA KAKGVSLLLP TDVVVADKFA PDANSKV--- -----VP-AA AIPDGWMG-L DIGPDSVKTF
AQK86959.1 QGLPVG---- -ASLVEEDKL ELATSLLEKA KEKGVSLLLP TDVVIADKFA PDANSQI--- -----VP-AS AIPDGWMG-L DIGPDSVASF
Zm00001d01 QGLSVG---- -ASLVEEDKL DLATSLLAKA KEKGVSLMLP TDVVIADKFA PDANSQI--- -----VP-AS AIPDGWMG-L DIGPDSIAAF
A8JC04_cPG RGLKVG---- -SSLVEDDKI ELAKKLEEMA KAKGVQLLLP TDVVVADKFD ANANTQT--- -----VP-IT AIPDGWMG-L DIGPDSVKTF
GAQ79801.1 RGLAVG---- -SSLVEEDKL ELAKELTKMA EEKGVELILP TDVVVADKFA EDAENKT--- -----VK-VE EIPEGWMG-L DIGPDSIKAF
sp_P18912. LGHDVG---- -KSLLEEDKI ELAKSFMEKA KEKGVRFYMP VDVVVADRFA NDANTKV--- -----VP-ID AIPADWSA-L DIGPKTRELY
AAC75963.1 QGHDVG---- -KSLYEADLV DEAKRLLTTC -----NIPVP SDVRVATEFS ETAPATL--- -----KS-VN DVKADEQI-L DIGDASAQEL
sp|P50315| AGHPVGYDID DANLYDEQWE ANSEKIESML EDHRDQITLA VDLAYEDEND DRAEQAV--- ---------D DIDEKRLSYL DVGSETLMEY
sp_P00560. ENTEIG---- -DSIFDKAGA EIVPKLMEKA KAKGVEVVLP VDFIIADAFS ADANTKT--- -----VTDKE GIPAGWQG-L DNGPESRKLF
NP_112467. KNMQIG---- -ASLFDEEGA TIVKEIMEKA EKNGVKIVFP VDFVTGDKFD ENAKVGQ--- -----ATIES GIPSGWMG-L DCGPESIKIN
sp|P07205. NNMEIG---- -ASLFDEEGA KIVKDIMAKA QKNGVRITFP VDFVTGDKFD ENAQVGK--- -----ATVAS GISPGWMG-L DCGPESNKNH
sp|P00558. NNMEIG---- -TSLFDEEGA KIVKDLMSKA EKNGVKITLP VDFVTADKFD ENAKTGQ--- -----ATVAS GIPAGWMG-L DCGPESSKKY
NP_032854. NNMEIG---- -TSLYDEEGA KIVKDLMSKA EKNGVKITLP VDFVTADKFD ENAKTGQ--- -----ATVAS GIPAGWMG-L DCGTESSKKY
Zm00001d03 FGMTVP---- -SQFIERNAV KEVLQIIQVA RDRNIPIYYP TDLWCLKNGD SEI-SGV--- -----IN-ST GQLAGWTP-A DIGPSTLEKI
tr_A0A1S4D LGLSVS---- -YRLMNHGVC KEAADLIQFS LDKRVQIVYP KDFWCVNVDT SK-KIEI--- -----FA-SH DIPDGWLP-V DLGPTSLDEI
XP_0165427 FGLAVP---- -MVLVEQESL EAAHMLVETA KSKGVQLVLP KDFWCLNDHY P-SQMKI--- -----FP-AN HIMDGWKP-V DVGPNTLEEM
XP_0063543 FGLPVP---- -MELVEQESL EAAHVLVEAA KARGVQIILP KDFWCLNDYY P-SQMKI--- -----FP-AN HIMDGWKP-V DVGPNTLEEM
XP_0258888 FGLPVP---- -MELVEQESL EAARVLVEAA KARGVQIILP KDFWCINDYY P-SRMKI--- -----FP-AN RIIDGWKP-V DVGPNTLGEM
XP_0042466 FGLPVP---- -MELVEQESL EAARVLVEAA KARGVQIILP KDFWCINDYY P-SRMKI--- -----FP-AN RIIDGWKP-V DVGPNTLGEM
KAF3623713 QGLSVG---- -SSLVEEDKL DLATSLLEKA KAKGVSLLLP SDVVIADKFA PDANSKI--- -----VP-AS AIPDGWMG-L DIGPDSVKTF
GAQ85336.1 LGWRTG---- -GTPTEAGST ERATELLELA EKEGVELVFP DDVVCGEAQV HSAKVKI--- -----CT-AR HVLHEWSA-M DIGPQTIDHY
A8JFT4_PGK RGVSVG---- -STQIEPDWL EPCRAMMARA GQRGTQLLLP GDVLWSSSLA APVDTGVQVL TLDCCTEESP CIPAGRYG-V DIGPETSAAF
```

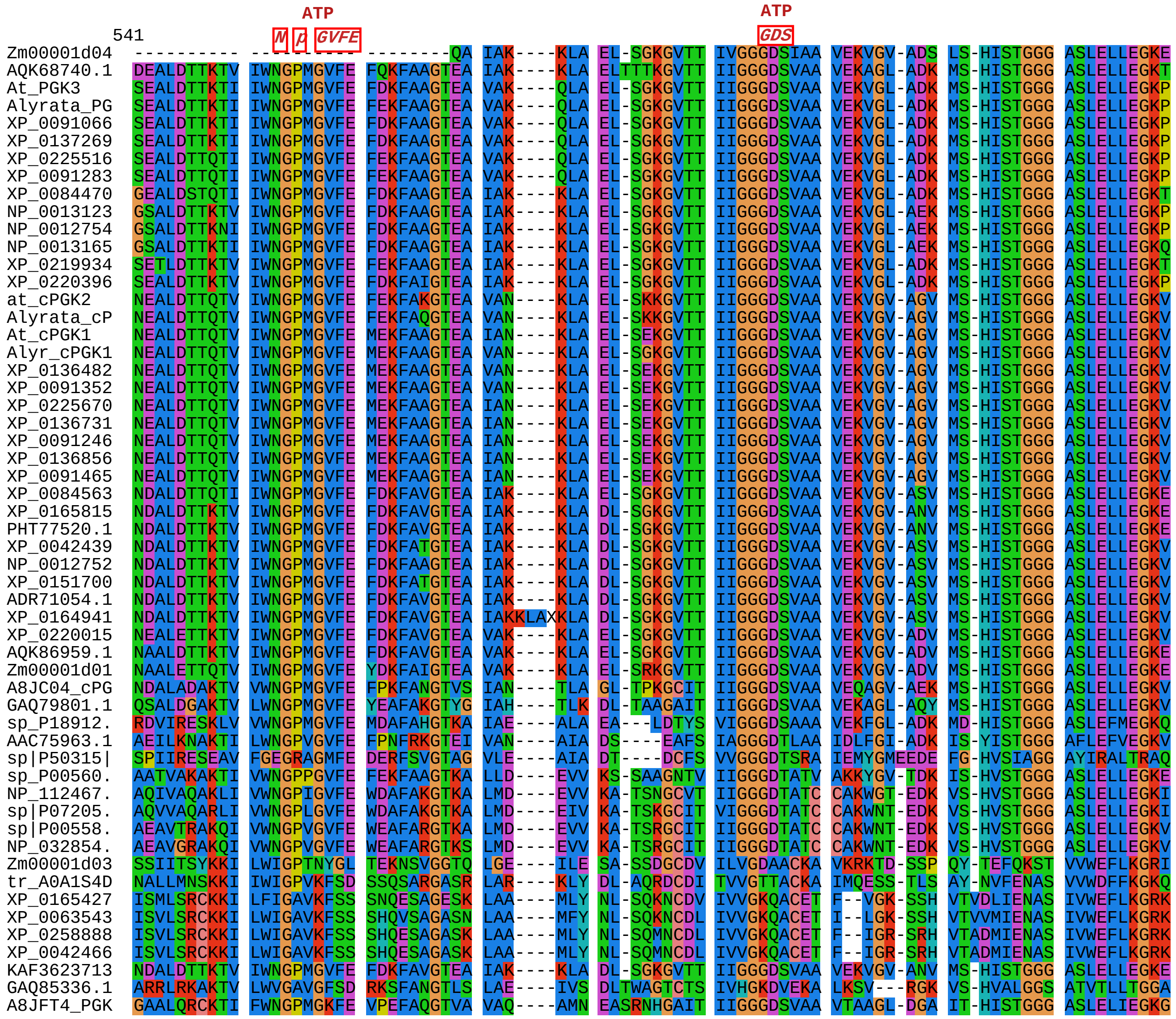
ATP
ATP
541
M Λ GVFE
GDS
Zm00001d04 ---------- ---------- ---------QA IAK----KLA EL-SGKGVTT IVGGGDSIAA VEKVGV-ADS LS-HISTGGG ASLELLEGKE
AQK68740.1 DEALDTTKTV IWNGPMGVFE FQKFAAGTEA IAK----KLA ELTTTKGVTT IIGGGDSVAA VEKAGL-ADK MS-HISTGGG ASLELLEGKT
At_PGK3 SEALDTTKTI IWNGPMGVFE FDKFAAGTEA VAK----QLA EL-SGKGVTT IIGGGDSVAA VEKVGL-ADK MS-HISTGGG ASLELLEGKP
Alyrata_PG SEALDTTKTI IWNGPMGVFE FEKFAAGTEA VAK----QLA EL-SGKGVTT IIGGGDSVAA VEKVGL-ADK MS-HISTGGG ASLELLEGKP
XP_0091066 SEALDTTKTI IWNGPMGVFE FDKFAAGTEA VAK----QLA EL-SGKGVTT IIGGGDSVAA VEKVGL-ADK MS-HISTGGG ASLELLEGKP
XP_0137269 SEALDTTKTI IWNGPMGVFE FDKFAAGTEA VAK----QLA EL-SGKGVTT IIGGGDSVAA VEKVGL-ADK MS-HISTGGG ASLELLEGKP
XP_0225516 SEALDTTQTI IWNGPMGVFE FEKFAAGTEA VAK----QLA EL-SGKGVTT IIGGGDSVAA VEKVGL-ADK MS-HISTGGG ASLELLEGKP
XP_0091283 SEALDTTQTI IWNGPMGVFE FEKFAAGTEA VAK----QLA EL-SGKGVTT IIGGGDSVAA VEKVGL-ADK MS-HISTGGG ASLELLEGKP
XP_0084470 GEALDSTQTI IWNGPMGVFE FDKFAAGTEA IAK----KLA EL-SGKGVTT IIGGGDSVAA VEKVGL-ADK MS-HISTGGG ASLELLEGKT
NP_0013123 GSALDTTKTV IWNGPMGVFE FDKFAAGTEA IAK----KLA EL-SGKGVTT IIGGGDSVAA VEKVGL-AEK MS-HISTGGG ASLELLEGKP
NP_0012754 GSALDTTKNI IWNGPMGVFE FDKFAAGTEA IAK----KLA EL-SGKGVTT IIGGGDSVAA VEKVGL-AEK MS-HISTGGG ASLELLEGKP
NP_0013165 GSALDTTKTI IWNGPMGVFE FDKFAAGTEA IAK----KLA EL-SGKGVTT IIGGGDSVAA VEKVGL-AEK MS-HISTGGG ASLELLEGKQ
XP_0219934 SETLDTTKTV IWNGPMGVFE FEKFAAGTEA IAK----KLA EL-SGKGVTT IIGGGDSVAA VEKVGL-ADK MS-HISTGGG ASLELLEGKT
XP_0220396 SEALDTTKTV IWNGPMGVFE FDKFAIGTEA IAK----KLA EL-SGKGVTT IIGGGDSVAA VEKVGL-ADK MS-HISTGGG ASLELLEGKP
at_cPGK2 NEALDTTQTV IWNGPMGVFE FEKFAKGTEA VAN----KLA EL-SKKGVTT IIGGGDSVAA VEKVGV-AGV MS-HISTGGG ASLELLEGKV
Alyrata_cP NEALDTTQTV IWNGPMGVFE FEKFAQGTEA VAN----KLA EL-SKKGVTT IIGGGDSVAA VEKVGV-AGV MS-HISTGGG ASLELLEGKV
At_cPGK1 NEALDTTQTV IWNGPMGVFE MEKFAAGTEA IAN----KLA EL-SEKGVTT IIGGGDSVAA VEKVGV-AGV MS-HISTGGG ASLELLEGKV
Alyr_cPGK1 NEALDTTQTV IWNGPMGVFE MEKFAAGTEA VAN----KLA EL-SGKGVTT IIGGGDSVAA VEKVGV-AGV MS-HISTGGG ASLELLEGKV
XP_0136482 NEALDTTQTV IWNGPMGVFE MEKFAAGTEA VAN----KLA EL-SEKGVTT IIGGGDSVAA VEKVGV-AGV MS-HISTGGG ASLELLEGKV
XP_0091352 NEALDTTQTV IWNGPMGVFE MEKFAAGTEA VAN----KLA EL-SEKGVTT IIGGGDSVAA VEKVGV-AGV MS-HISTGGG ASLELLEGKV
XP_0225670 NEALDTTQTV IWNGPMGVFE MEKFAAGTEA IAN----KLA EL-SEKGVTT IIGGGDSVAA VEKVGV-AGV MS-HISTGGG ASLELLEGKV
XP_0136731 NEALDTTQTV IWNGPMGVFE MEKFAAGTEA IAN----KLA EL-SEKGVTT IIGGGDSVAA VEKVGV-AGV MS-HISTGGG ASLELLEGKV
XP_0091246 NEALDTTQTV IWNGPMGVFE MEKFAAGTEA IAN----KLA EL-SEKGVTT IIGGGDSVAA VEKVGV-AGV MS-HISTGGG ASLELLEGKV
XP_0136856 NEALDTTQTV IWNGPMGVFE MEKFAAGTEA IAN----KLA EL-SEKGVTT IIGGGDSVAA VEKVGV-AGV MS-HISTGGG ASLELLEGKV
XP_0091465 NEALDTTQTV IWNGPMGVFE MEKFAAGTEA IAN----KLA EL-SEKGVTT IIGGGDSVAA VEKVGV-AGV MS-HISTGGG ASLELLEGKV
XP_0084563 NDALDTTQTI IWNGPMGVFE FDKFAAGTEA IAK----KLA EL-SGKGVTT IIGGGDSVAA VEKVGV-ASV MS-HISTGGG ASLELLEGKV
XP_0165815 NDALDTTKTV IWNGPMGVFE FDKFAVGTEA IAK----KLA DL-SGKGVTT IIGGGDSVAA VEKVGV-ANV MS-HISTGGG ASLELLEGKE
PHT77520.1 NDALDTTKTV IWNGPMGVFE FDKFAVGTEA IAK----KLA DL-SGKGVTT IIGGGDSVAA VEKVGV-ANV MS-HISTGGG ASLELLEGKE
XP_0042439 NDALDTTKTV IWNGPMGVFE FDKFATGTEA IAK----KLA DL-SGKGVTT IIGGGDSVAA VEKVGV-ASV MS-HISTGGG ASLELLEGKV
NP_0012752 NDALDTTKTV IWNGPMGVFE FDKFAAGTEA IAK----KLA DL-SGKGVTT IIGGGDSVAA VEKVGV-ASV MS-HISTGGG ASLELLEGKV
XP_0151700 NDALDTTKTV IWNGPMGVFE FDKFATGTEA IAK----KLA DL-SGKGVTT IIGGGDSVAA VEKVGV-ASV MS-HISTGGG ASLELLEGKV
ADR71054.1 NDALDTTKTV IWNGPMGVFE FDKFAVGTEA IAK----KLA DL-SGKGVTT IIGGGDSVAA VEKVGV-ASV MS-HISTGGG ASLELLEGKV
XP_0164941 NDALDTTKTV IWNGPMGVFE FDKFAVGTEA IAKKLAXKLA DL-SGKGVTT IIGGGDSVAA VEKVGV-ASV MS-HISTGGG ASLELLEGKV
XP_0220015 NEALETTKTV IWNGPMGVFE FDKFAVGTEA VAK----KLA EL-SGKGVTT IIGGGDSVAA VEKVGV-ADV MS-HISTGGG ASLELLEGKV
AQK86959.1 NAALDTTKTV IWNGPMGVFE FDKFAAGTEA VAK----KLA EL-SGKGVTT IIGGGDSVAA VEKVGV-ADV MS-HISTGGG ASLELLEGKE
Zm00001d01 NAALETTQTV IWNGPMGVFE YDKFAIGTEA VAK----KLA EL-SRKGVTT IIGGGDSVAA VEKVGV-ADV MS-HISTGGG ASLELLEGKE
A8JC04_cPG NDALADAKTV VWNGPMGVFE FPKFANGTVS IAN----TLA GL-TPKGCIT IIGGGDSVAA VEQAGV-AEK MS-HISTGGG ASLELLEGKV
GAQ79801.1 QSALDGAKTV LWNGPMGVFE YEAFAKGTYG IAH----TLK DL-TAAGAIT IIGGGDSVAA VEKAGL-AQY MS-HISTGGG ASLELLEGKV
sp_P18912. RDVIRESKLV VWNGPMGVFE MDAFAHGTKA IAE----ALA EA---LDTYS VIGGGDSAAA VEKFGL-ADK MD-HISTGGG ASLEFMEGKQ
AAC75963.1 AEILKNAKTI LWNGPVGVFE FPNFRKGTEI VAN----AIA DS----EAFS IAGGGDTLAA IDLFGI-ADK IS-YISTGGG AFLEFVEGKV
sp|P50315| SPIIRESEAV FGEGRAGMFE DERFSVGTAG VLE----AIA DT----DCFS VVGGGDTSRA IEMYGMEEDE FG-HVSIAGG AYIRALTRAQ
sp_P00560. AATVAKAKTI VWNGPPGVFE FEKFAAGTKA LLD----EVV KS-SAAGNTV IIGGGDTATV AKKYGV-TDK IS-HVSTGGG ASLELLEGKE
NP_112467. AQIVAQAKLI VWNGPIGVFE WDAFAKGTKA LMD----EVV KA-TSNGCVT IIGGGDTATC CAKWGT-EDK VS-HVSTGGG ASLELLEGKI
sp|P07205. AQVVAQARLI VWNGPLGVFE WDAFAKGTKA LMD----EIV KA-TSKGCIT VIGGGDTATC CAKWNT-EDK VS-HVSTGGG ASLELLEGKI
sp|P00558. AEAVTRAKQI VWNGPVGVFE WEAFARGTKA LMD----EVV KA-TSRGCIT IIGGGDTATC CAKWNT-EDK VS-HVSTGGG ASLELLEGKV
NP_032854. AEAVGRAKQI VWNGPVGVFE WEAFARGTKS LMD----EVV KA-TSRGCIT IIGGGDTATC CAKWNT-EDK VS-HVSTGGG ASLELLEGKV
Zm00001d03 SSIITSYKKI LWIGPTNYGL TEKNSVGGTQ LGE----ILE SA-SSDGCDV ILVGDAACKA VKRKTD-SSP QY-TEFQKST VVWEFLKGRI
tr_A0A1S4D NALLMNSKKI IWIGPVKFSD SSQSARGASR LAR----KLY DL-AQRDCDI TVVGTTACKA IMQESS-TLS AY-NVFENAS VVWDFFKGKQ
XP_0165427 ISMLSRCKKI LFIGAVKFSS SNQESAGESK LAA----MLY NL-SQKNCDV IVVGKQACET F--VGK-SSH VTVDLIENAS IVWEFLKGRK
XP_0063543 ISVLSRCKKI LWIGAVKFSS SHQVSAGASN LAA----MFY NL-SQKNCDL IVVGKQACET I--LGK-SSH VTVVMIENAS IVWEFLKGRK
XP_0258888 ISVLSRCKKI LWIGAVKFSS SHQESAGASK LAA----MLY NL-SQMNCDL IVVGKQACET F--IGR-SRH VTADMIENAS IVWEFLKGRK
XP_0042466 ISVLSRCKKI LWIGAVKFSS SHQESAGASK LAA----MLY NL-SQMNCDL IVVGKQACET F--IGR-SRH VTADMIENAS IVWEFLKGRK
KAF3623713 NDALDTTKTV IWNGPMGVFE FDKFAVGTEA IAK----KLA DL-SGKGVTT IIGGGDSVAA VEKVGV-ANV MS-HISTGGG ASLELLEGKE
GAQ85336.1 ARRLRKAKTV LWVGAVGFSD RKSFANGTLS LAE----IVS DLTWAGTCTS IVHGKDVEKA LKSV---RGK VS-HVALGGS ATVTLLTGGA
A8JFT4_PGK GAALQRCKTI FWNGPMGKFE VPEFAQGTVA VAQ----AMN EASRNHGAIT IIGGGDSVAA VTAAGL-DGA IT-HISTGGG ASLELIEGKG

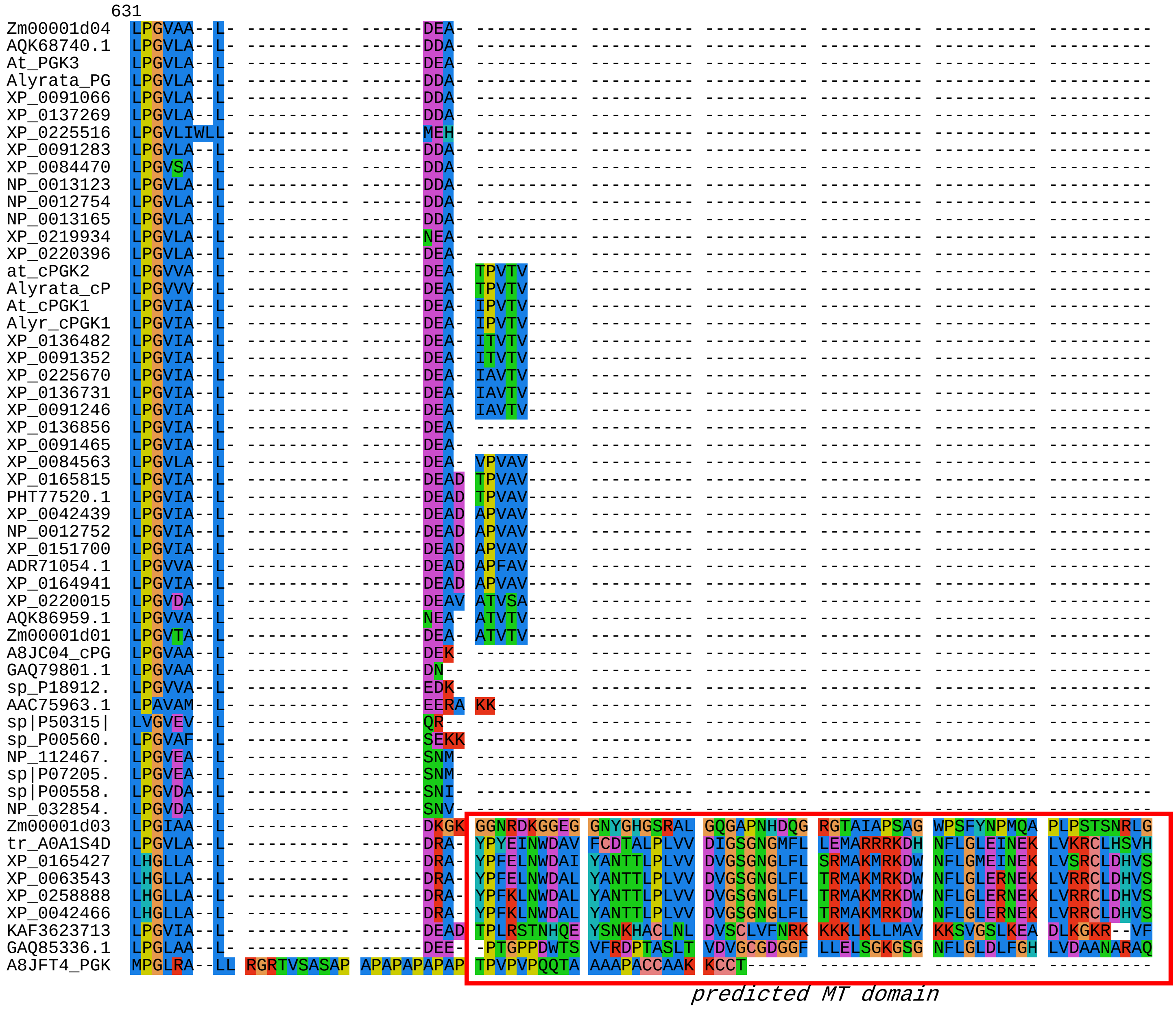
631
Zm00001d04 LPGVAA--L- ---------- ------DEA- ---------- ---------- ---------- ---------- ---------- ----------
AQK68740.1 LPGVLA--L- ---------- ------DDA- ---------- ---------- ---------- ---------- ---------- ----------
At_PGK3 LPGVLA--L- ---------- ------DEA- ---------- ---------- ---------- ---------- ---------- ----------
Alyrata_PG LPGVLA--L- ---------- ------DDA- ---------- ---------- ---------- ---------- ---------- ----------
XP_0091066 LPGVLA--L- ---------- ------DDA- ---------- ---------- ---------- ---------- ---------- ----------
XP_0137269 LPGVLA--L- ---------- ------DDA- ---------- ---------- ---------- ---------- ---------- ----------
XP_0225516 LPGVLIWLL- ---------- ------MEH- ---------- ---------- ---------- ---------- ---------- ----------
XP_0091283 LPGVLA--L- ---------- ------DDA- ---------- ---------- ---------- ---------- ---------- ----------
XP_0084470 LPGVSA--L- ---------- ------DDA- ---------- ---------- ---------- ---------- ---------- ----------
NP_0013123 LPGVLA--L- ---------- ------DDA- ---------- ---------- ---------- ---------- ---------- ----------
NP_0012754 LPGVLA--L- ---------- ------DDA- ---------- ---------- ---------- ---------- ---------- ----------
NP_0013165 LPGVLA--L- ---------- ------DDA- ---------- ---------- ---------- ---------- ---------- ----------
XP_0219934 LPGVLA--L- ---------- ------NEA- ---------- ---------- ---------- ---------- ---------- ----------
XP_0220396 LPGVLA--L- ---------- ------DEA- ---------- ---------- ---------- ---------- ---------- ----------
at_cPGK2 LPGVVA--L- ---------- ------DEA- TPVTV----- ---------- ---------- ---------- ---------- ----------
Alyrata_cP LPGVVV--L- ---------- ------DEA- TPVTV----- ---------- ---------- ---------- ---------- ----------
At_cPGK1 LPGVIA--L- ---------- ------DEA- IPVTV----- ---------- ---------- ---------- ---------- ----------
Alyr_cPGK1 LPGVIA--L- ---------- ------DEA- IPVTV----- ---------- ---------- ---------- ---------- ----------
XP_0136482 LPGVIA--L- ---------- ------DEA- ITVTV----- ---------- ---------- ---------- ---------- ----------
XP_0091352 LPGVIA--L- ---------- ------DEA- ITVTV----- ---------- ---------- ---------- ---------- ----------
XP_0225670 LPGVIA--L- ---------- ------DEA- IAVTV----- ---------- ---------- ---------- ---------- ----------
XP_0136731 LPGVIA--L- ---------- ------DEA- IAVTV----- ---------- ---------- ---------- ---------- ----------
XP_0091246 LPGVIA--L- ---------- ------DEA- IAVTV----- ---------- ---------- ---------- ---------- ----------
XP_0136856 LPGVIA--L- ---------- ------DEA- ---------- ---------- ---------- ---------- ---------- ----------
XP_0091465 LPGVIA--L- ---------- ------DEA- ---------- ---------- ---------- ---------- ---------- ----------
XP_0084563 LPGVLA--L- ---------- ------DEA- VPVAV----- ---------- ---------- ---------- ---------- ----------
XP_0165815 LPGVIA--L- ---------- ------DEAD TPVAV----- ---------- ---------- ---------- ---------- ----------
PHT77520.1 LPGVIA--L- ---------- ------DEAD TPVAV----- ---------- ---------- ---------- ---------- ----------
XP_0042439 LPGVIA--L- ---------- ------DEAD APVAV----- ---------- ---------- ---------- ---------- ----------
NP_0012752 LPGVIA--L- ---------- ------DEAD APVAV----- ---------- ---------- ---------- ---------- ----------
XP_0151700 LPGVIA--L- ---------- ------DEAD APVAV----- ---------- ---------- ---------- ---------- ----------
ADR71054.1 LPGVVA--L- ---------- ------DEAD APFAV----- ---------- ---------- ---------- ---------- ----------
XP_0164941 LPGVIA--L- ---------- ------DEAD APVAV----- ---------- ---------- ---------- ---------- ----------
XP_0220015 LPGVDA--L- ---------- ------DEAV ATVSA----- ---------- ---------- ---------- ---------- ----------
AQK86959.1 LPGVVA--L- ---------- ------NEA- ATVTV----- ---------- ---------- ---------- ---------- ----------
Zm00001d01 LPGVTA--L- ---------- ------DEA- ATVTV----- ---------- ---------- ---------- ---------- ----------
A8JC04_cPG LPGVAA--L- ---------- ------DEK- ---------- ---------- ---------- ---------- ---------- ----------
GAQ79801.1 LPGVAA--L- ---------- ------DN-- ---------- ---------- ---------- ---------- ---------- ----------
sp_P18912. LPGVVA--L- ---------- ------EDK- ---------- ---------- ---------- ---------- ---------- ----------
AAC75963.1 LPAVAM--L- ---------- ------EERA KK-------- ---------- ---------- ---------- ---------- ----------
sp|P50315| LVGVEV--L- ---------- ------QR-- ---------- ---------- ---------- ---------- ---------- ----------
sp_P00560. LPGVAF--L- ---------- ------SEKK ---------- ---------- ---------- ---------- ---------- ----------
NP_112467. LPGVEA--L- ---------- ------SNM- ---------- ---------- ---------- ---------- ---------- ----------
sp|P07205. LPGVEA--L- ---------- ------SNM- ---------- ---------- ---------- ---------- ---------- ----------
sp|P00558. LPGVDA--L- ---------- ------SNI- ---------- ---------- ---------- ---------- ---------- ----------
NP_032854. LPGVDA--L- ---------- ------SNV- ---------- ---------- ---------- ---------- ---------- ----------
Zm00001d03 LPGIAA--L- ---------- ------DKGK GGNRDKGGEG GNYGHGSRAL GQGAPNHDQG RGTAIAPSAG WPSFYNPMQA PLPSTSNRLG
tr_A0A1S4D LPGVLA--L- ---------- ------DRA- YPYEINWDAV FCDTALPLVV DIGSGNGMFL LEMARRRKDH NFLGLEINEK LVKRCLHSVH
XP_0165427 LHGLLA--L- ---------- ------DRA- YPFELNWDAI YANTTLPLVV DVGSGNGLFL SRMAKMRKDW NFLGMEINEK LVSRCLDHVS
XP_0063543 LHGLLA--L- ---------- ------DRA- YPFELNWDAL YANTTLPLVV DVGSGNGLFL TRMAKMRKDW NFLGLERNEK LVRRCLDHVS
XP_0258888 LHGLLA--L- ---------- ------DRA- YPFKLNWDAL YANTTLPLVV DVGSGNGLFL TRMAKMRKDW NFLGLERNEK LVRRCLDHVS
XP_0042466 LHGLLA--L- ---------- ------DRA- YPFKLNWDAL YANTTLPLVV DVGSGNGLFL TRMAKMRKDW NFLGLERNEK LVRRCLDHVS
KAF3623713 LPGVIA--L- ---------- ------DEAD TPLRSTNHQE YSNKHACLNL DVSCLVFNRK KKKLKLLMAV KKSVGSLKEA DLKGKR--VF
GAQ85336.1 LPGLAA--L- ---------- ------DEE- -PTGPPDWTS VFRDPTASLT VDVGCGDGGF LLELSGKGSG NFLGLDLFGH LVDAANARAQ
A8JFT4_PGK MPGLRA--LL RGRTVSASAP APAPAPAPAP TPVPVPQQTA AAAPACCAAK KCCT------ ---------- ---------- ----------
predicted MT domain

```
            721
Zm00001d04  ---------- ---------- ---------- ---------- ---------- ---------- ---------- ---------- ----------
AQK68740.1  ---------- ---------- ---------- ---------- ---------- ---------- ---------- ---------- ----------
At_PGK3     ---------- ---------- ---------- ---------- ---------- ---------- ---------- ---------- ----------
Alyrata_PG  ---------- ---------- ---------- ---------- ---------- ---------- ---------- ---------- ----------
XP_0091066  ---------- ---------- ---------- ---------- ---------- ---------- ---------- ---------- ----------
XP_0137269  ---------- ---------- ---------- ---------- ---------- ---------- ---------- ---------- ----------
XP_0225516  ---------- ---------- ---------- ---------- ---------- ---------- ---------- ---------- ----------
XP_0091283  ---------- ---------- ---------- ---------- ---------- ---------- ---------- ---------- ----------
XP_0084470  ---------- ---------- ---------- ---------- ---------- ---------- ---------- ---------- ----------
NP_0013123  ---------- ---------- ---------- ---------- ---------- ---------- ---------- ---------- ----------
NP_0012754  ---------- ---------- ---------- ---------- ---------- ---------- ---------- ---------- ----------
NP_0013165  ---------- ---------- ---------- ---------- ---------- ---------- ---------- ---------- ----------
XP_0219934  ---------- ---------- ---------- ---------- ---------- ---------- ---------- ---------- ----------
XP_0220396  ---------- ---------- ---------- ---------- ---------- ---------- ---------- ---------- ----------
at_cPGK2    ---------- ---------- ---------- ---------- ---------- ---------- ---------- ---------- ----------
Alyrata_cP  ---------- ---------- ---------- ---------- ---------- ---------- ---------- ---------- ----------
At_cPGK1    ---------- ---------- ---------- ---------- ---------- ---------- ---------- ---------- ----------
Alyr_cPGK1  ---------- ---------- ---------- ---------- ---------- ---------- ---------- ---------- ----------
XP_0136482  ---------- ---------- ---------- ---------- ---------- ---------- ---------- ---------- ----------
XP_0091352  ---------- ---------- ---------- ---------- ---------- ---------- ---------- ---------- ----------
XP_0225670  ---------- ---------- ---------- ---------- ---------- ---------- ---------- ---------- ----------
XP_0136731  ---------- ---------- ---------- ---------- ---------- ---------- ---------- ---------- ----------
XP_0091246  ---------- ---------- ---------- ---------- ---------- ---------- ---------- ---------- ----------
XP_0136856  ---------- ---------- ---------- ---------- ---------- ---------- ---------- ---------- ----------
XP_0091465  ---------- ---------- ---------- ---------- ---------- ---------- ---------- ---------- ----------
XP_0084563  ---------- ---------- ---------- ---------- ---------- ---------- ---------- ---------- ----------
XP_0165815  ---------- ---------- ---------- ---------- ---------- ---------- ---------- ---------- ----------
PHT77520.1  ---------- ---------- ---------- ---------- ---------- ---------- ---------- ---------- ----------
XP_0042439  ---------- ---------- ---------- ---------- ---------- ---------- ---------- ---------- ----------
NP_0012752  ---------- ---------- ---------- ---------- ---------- ---------- ---------- ---------- ----------
XP_0151700  ---------- ---------- ---------- ---------- ---------- ---------- ---------- ---------- ----------
ADR71054.1  ---------- ---------- ---------- ---------- ---------- ---------- ---------- ---------- ----------
XP_0164941  ---------- ---------- ---------- ---------- ---------- ---------- ---------- ---------- ----------
XP_0220015  ---------- ---------- ---------- ---------- ---------- ---------- ---------- ---------- ----------
AQK86959.1  ---------- ---------- ---------- ---------- ---------- ---------- ---------- ---------- ----------
Zm00001d01  ---------- ---------- ---------- ---------- ---------- ---------- ---------- ---------- ----------
A8JC04_cPG  ---------- ---------- ---------- ---------- ---------- ---------- ---------- ---------- ----------
GAQ79801.1  ---------- ---------- ---------- ---------- ---------- ---------- ---------- ---------- ----------
sp_P18912.  ---------- ---------- ---------- ---------- ---------- ---------- ---------- ---------- ----------
AAC75963.1  ---------- ---------- ---------- ---------- ---------- ---------- ---------- ---------- ----------
sp|P50315|  ---------- ---------- ---------- ---------- ---------- ---------- ---------- ---------- ----------
sp_P00560.  ---------- ---------- ---------- ---------- ---------- ---------- ---------- ---------- ----------
NP_112467.  ---------- ---------- ---------- ---------- ---------- ---------- ---------- ---------- ----------
sp|P07205.  ---------- ---------- ---------- ---------- ---------- ---------- ---------- ---------- ----------
sp|P00558.  ---------- ---------- ---------- ---------- ---------- ---------- ---------- ---------- ----------
NP_032854.  ---------- ---------- ---------- ---------- ---------- ---------- ---------- ---------- ----------
Zm00001d03  GSTSSSVATS VVLCPRCFRP AKASD----- ---------- ---------- ---------- ---------- ---------- ----------
tr_A0A1S4D  QLEMKNGHFI ATNATSTFRS IVCSYPGELV LVSIQCPNPD FNKPEHRWRM LQRSLVEAVA DLLASNGKVF LQSDVEAVAL RMREAFLLYG
XP_0165427  QSGMTNGYFI AANATSTFRS IVSSYPGDLV LVSIQCPNPD FNKTEHRWRM VQRSLVEALA DLLASDGKVF LQSDVKEVVV RMREEFMKYG
XP_0063543  QSGMTNGYFI ATNATSTFRS IVSSYPGDLV LVSIQCPNPD FNKTEHRWRM VQRSLVEAIA DLVAPDGKVF LQSDVKEVAV RMKKEFMKYG
XP_0258888  QSGMTNGNEC HIN------- ---------- -IPIHC---- ---------- ---------- ---------- ---------- ----------
XP_0042466  QSGMTNGYFI ATNATSTFRF IVSSYPGDLV LVSIQCPNPD FNRTEHRWRM VQRSLVEAIA DLVAPDGKVF LQSDVKEVAV RMKKEFMNYG
KAF3623713  VRVDLNVPLD DNFNITDDTR IRAAVPTIKY LMQNGARVIL ASHLGRPKGV TPKYSLKPLI PRLSELLGVE VKMANDSVGP EVEKLVAEIP
GAQ85336.1  REGRQNLYYV AANATTSLGP MLSTYPGALQ LVTIQCPTPQ FEARGLGRLV LRQPLVEAIT EKLVTGGRLF VQSDVNDVAS SMLEEIQKHG
A8JFT4_PGK  ---------- ---------- ---------- ---------- ---------- ---------- ---------- ---------- ----------
```

predicted MT domain

```
             811
Zm00001d04  ---------- ---------- ---------- ---------- ---------- ---------- ---------- ---------- ---------- ----------
AQK68740.1  ---------- ---------- ---------- ---------- ---------- ---------- ---------- ---------- ---------- ----------
At_PGK3     ---------- ---------- ---------- ---------- ---------- ---------- ---------- ---------- ---------- ----------
Alyrata_PG  ---------- ---------- ---------- ---------- ---------- ---------- ---------- ---------- ---------- ----------
XP_0091066  ---------- ---------- ---------- ---------- ---------- ---------- ---------- ---------- ---------- ----------
XP_0137269  ---------- ---------- ---------- ---------- ---------- ---------- ---------- ---------- ---------- ----------
XP_0225516  ---------- ---------- ---------- ---------- ---------- ---------- ---------- ---------- ---------- ----------
XP_0091283  ---------- ---------- ---------- ---------- ---------- ---------- ---------- ---------- ---------- ----------
XP_0084470  ---------- ---------- ---------- ---------- ---------- ---------- ---------- ---------- ---------- ----------
NP_0013123  ---------- ---------- ---------- ---------- ---------- ---------- ---------- ---------- ---------- ----------
NP_0012754  ---------- ---------- ---------- ---------- ---------- ---------- ---------- ---------- ---------- ----------
NP_0013165  ---------- ---------- ---------- ---------- ---------- ---------- ---------- ---------- ---------- ----------
XP_0219934  ---------- ---------- ---------- ---------- ---------- ---------- ---------- ---------- ---------- ----------
XP_0220396  ---------- ---------- ---------- ---------- ---------- ---------- ---------- ---------- ---------- ----------
at_cPGK2    ---------- ---------- ---------- ---------- ---------- ---------- ---------- ---------- ---------- ----------
Alyrata_cP  ---------- ---------- ---------- ---------- ---------- ---------- ---------- ---------- ---------- ----------
At_cPGK1    ---------- ---------- ---------- ---------- ---------- ---------- ---------- ---------- ---------- ----------
Alyr_cPGK1  ---------- ---------- ---------- ---------- ---------- ---------- ---------- ---------- ---------- ----------
XP_0136482  ---------- ---------- ---------- ---------- ---------- ---------- ---------- ---------- ---------- ----------
XP_0091352  ---------- ---------- ---------- ---------- ---------- ---------- ---------- ---------- ---------- ----------
XP_0225670  ---------- ---------- ---------- ---------- ---------- ---------- ---------- ---------- ---------- ----------
XP_0136731  ---------- ---------- ---------- ---------- ---------- ---------- ---------- ---------- ---------- ----------
XP_0091246  ---------- ---------- ---------- ---------- ---------- ---------- ---------- ---------- ---------- ----------
XP_0136856  ---------- ---------- ---------- ---------- ---------- ---------- ---------- ---------- ---------- ----------
XP_0091465  ---------- ---------- ---------- ---------- ---------- ---------- ---------- ---------- ---------- ----------
XP_0084563  ---------- ---------- ---------- ---------- ---------- ---------- ---------- ---------- ---------- ----------
XP_0165815  ---------- ---------- ---------- ---------- ---------- ---------- ---------- ---------- ---------- ----------
PHT77520.1  ---------- ---------- ---------- ---------- ---------- ---------- ---------- ---------- ---------- ----------
XP_0042439  ---------- ---------- ---------- ---------- ---------- ---------- ---------- ---------- ---------- ----------
NP_0012752  ---------- ---------- ---------- ---------- ---------- ---------- ---------- ---------- ---------- ----------
XP_0151700  ---------- ---------- ---------- ---------- ---------- ---------- ---------- ---------- ---------- ----------
ADR71054.1  ---------- ---------- ---------- ---------- ---------- ---------- ---------- ---------- ---------- ----------
XP_0164941  ---------- ---------- ---------- ---------- ---------- ---------- ---------- ---------- ---------- ----------
XP_0220015  ---------- ---------- ---------- ---------- ---------- ---------- ---------- ---------- ---------- ----------
AQK86959.1  ---------- ---------- ---------- ---------- ---------- ---------- ---------- ---------- ---------- ----------
Zm00001d01  ---------- ---------- ---------- ---------- ---------- ---------- ---------- ---------- ---------- ----------
A8JC04_cPG  ---------- ---------- ---------- ---------- ---------- ---------- ---------- ---------- ---------- ----------
GAQ79801.1  ---------- ---------- ---------- ---------- ---------- ---------- ---------- ---------- ---------- ----------
sp_P18912.  ---------- ---------- ---------- ---------- ---------- ---------- ---------- ---------- ---------- ----------
AAC75963.1  ---------- ---------- ---------- ---------- ---------- ---------- ---------- ---------- ---------- ----------
sp|P50315|  ---------- ---------- ---------- ---------- ---------- ---------- ---------- ---------- ---------- ----------
sp_P00560.  ---------- ---------- ---------- ---------- ---------- ---------- ---------- ---------- ---------- ----------
NP_112467.  ---------- ---------- ---------- ---------- ---------- ---------- ---------- ---------- ---------- ----------
sp|P07205.  ---------- ---------- ---------- ---------- ---------- ---------- ---------- ---------- ---------- ----------
sp|P00558.  ---------- ---------- ---------- ---------- ---------- ---------- ---------- ---------- ---------- ----------
NP_032854.  ---------- ---------- ---------- ---------- ---------- ---------- ---------- ---------- ---------- ----------
Zm00001d03  ---------- ---------- ---------- ---------- ---------- ---------- ---------- ---------- ---------- ----------
tr_A0A1S4D  KGKLGVLQEQ FGEEWLSENP FGVRSDWEQH VLDRGHPMFR LMLSNSTTNK SFN------- ---------- ---------- ---------- ----------
XP_0165427  KGKLTVMHDL EDSTSHRDEW LKENPFGIRS DWEQHVIDRG APMYRLLLLK SSPSG----- ---------- ---------- ---------- ----------
XP_0063543  KGKLTVMHDL EDITSHQDGW LNENPFGIRS DWEQHVIERG APMYRLLLLK SSPSG----- ---------- ---------- ---------- ----------
XP_0258888  ---------- ---------- -------FQL PW-------- ---------- ---------- ---------- ---------- ---------- ----------
XP_0042466  KGKLTVMHDS EDITSQQDGW LNENPFGIRS DWEQHVIERG APMYRLLLLK SSSSG----- ---------- ---------- ---------- ----------
KAF3623713  EGGVVLLENV RFYKEEEKND PEFSKKLASL ADLYVNDAFG TAHRAHASTE GVAKVLKPAV AGFLMQKELD YLVGAVSNPQ KPFAAIVGGS
GAQ85336.1  RGRLVNDEGF QGPGSSPLTL SSWKINPFGI PTQWETHALS KGRDMFRLMY RKH------- ---------- ---------- ---------- ----------
A8JFT4_PGK  ---------- ---------- ---------- ---------- ---------- ---------- ---------- ---------- ---------- ----------
```

predicted MT domain

```
             901
Zm00001d04  ---------- ---------- ---------- ---------- ---------- ---------- ---------- ---------- ----------
AQK68740.1  ---------- ---------- ---------- ---------- ---------- ---------- ---------- ---------- ----------
At_PGK3     ---------- ---------- ---------- ---------- ---------- ---------- ---------- ---------- ----------
Alyrata_PG  ---------- ---------- ---------- ---------- ---------- ---------- ---------- ---------- ----------
XP_0091066  ---------- ---------- ---------- ---------- ---------- ---------- ---------- ---------- ----------
XP_0137269  ---------- ---------- ---------- ---------- ---------- ---------- ---------- ---------- ----------
XP_0225516  ---------- ---------- ---------- ---------- ---------- ---------- ---------- ---------- ----------
XP_0091283  ---------- ---------- ---------- ---------- ---------- ---------- ---------- ---------- ----------
XP_0084470  ---------- ---------- ---------- ---------- ---------- ---------- ---------- ---------- ----------
NP_0013123  ---------- ---------- ---------- ---------- ---------- ---------- ---------- ---------- ----------
NP_0012754  ---------- ---------- ---------- ---------- ---------- ---------- ---------- ---------- ----------
NP_0013165  ---------- ---------- ---------- ---------- ---------- ---------- ---------- ---------- ----------
XP_0219934  ---------- ---------- ---------- ---------- ---------- ---------- ---------- ---------- ----------
XP_0220396  ---------- ---------- ---------- ---------- ---------- ---------- ---------- ---------- ----------
at_cPGK2    ---------- ---------- ---------- ---------- ---------- ---------- ---------- ---------- ----------
Alyrata_cP  ---------- ---------- ---------- ---------- ---------- ---------- ---------- ---------- ----------
At_cPGK1    ---------- ---------- ---------- ---------- ---------- ---------- ---------- ---------- ----------
Alyr_cPGK1  ---------- ---------- ---------- ---------- ---------- ---------- ---------- ---------- ----------
XP_0136482  ---------- ---------- ---------- ---------- ---------- ---------- ---------- ---------- ----------
XP_0091352  ---------- ---------- ---------- ---------- ---------- ---------- ---------- ---------- ----------
XP_0225670  ---------- ---------- ---------- ---------- ---------- ---------- ---------- ---------- ----------
XP_0136731  ---------- ---------- ---------- ---------- ---------- ---------- ---------- ---------- ----------
XP_0091246  ---------- ---------- ---------- ---------- ---------- ---------- ---------- ---------- ----------
XP_0136856  ---------- ---------- ---------- ---------- ---------- ---------- ---------- ---------- ----------
XP_0091465  ---------- ---------- ---------- ---------- ---------- ---------- ---------- ---------- ----------
XP_0084563  ---------- ---------- ---------- ---------- ---------- ---------- ---------- ---------- ----------
XP_0165815  ---------- ---------- ---------- ---------- ---------- ---------- ---------- ---------- ----------
PHT77520.1  ---------- ---------- ---------- ---------- ---------- ---------- ---------- ---------- ----------
XP_0042439  ---------- ---------- ---------- ---------- ---------- ---------- ---------- ---------- ----------
NP_0012752  ---------- ---------- ---------- ---------- ---------- ---------- ---------- ---------- ----------
XP_0151700  ---------- ---------- ---------- ---------- ---------- ---------- ---------- ---------- ----------
ADR71054.1  ---------- ---------- ---------- ---------- ---------- ---------- ---------- ---------- ----------
XP_0164941  ---------- ---------- ---------- ---------- ---------- ---------- ---------- ---------- ----------
XP_0220015  ---------- ---------- ---------- ---------- ---------- ---------- ---------- ---------- ----------
AQK86959.1  ---------- ---------- ---------- ---------- ---------- ---------- ---------- ---------- ----------
Zm00001d01  ---------- ---------- ---------- ---------- ---------- ---------- ---------- ---------- ----------
A8JC04_cPG  ---------- ---------- ---------- ---------- ---------- ---------- ---------- ---------- ----------
GAQ79801.1  ---------- ---------- ---------- ---------- ---------- ---------- ---------- ---------- ----------
sp_P18912.  ---------- ---------- ---------- ---------- ---------- ---------- ---------- ---------- ----------
AAC75963.1  ---------- ---------- ---------- ---------- ---------- ---------- ---------- ---------- ----------
sp|P50315|  ---------- ---------- ---------- ---------- ---------- ---------- ---------- ---------- ----------
sp_P00560.  ---------- ---------- ---------- ---------- ---------- ---------- ---------- ---------- ----------
NP_112467.  ---------- ---------- ---------- ---------- ---------- ---------- ---------- ---------- ----------
sp|P07205.  ---------- ---------- ---------- ---------- ---------- ---------- ---------- ---------- ----------
sp|P00558.  ---------- ---------- ---------- ---------- ---------- ---------- ---------- ---------- ----------
NP_032854.  ---------- ---------- ---------- ---------- ---------- ---------- ---------- ---------- ----------
Zm00001d03  ---------- ---------- ---------- ---------- ---------- ---------- ---------- ---------- ----------
tr_A0A1S4D  ---------- ---------- ---------- ---------- ---------- ---------- ---------- ---------- ----------
XP_0165427  ---------- ---------- ---------- ---------- ---------- ---------- ---------- ---------- ----------
XP_0063543  ---------- ---------- ---------- ---------- ---------- ---------- ---------- ---------- ----------
XP_0258888  ---------- ---------- ---------- ---------- ---------- ---------- ---------- ---------- ----------
XP_0042466  ---------- ---------- ---------- ---------- ---------- ---------- ---------- ---------- ----------
KAF3623713  KVSSKIGVIE SLLEKVDVLL LGGGMIFTFY KAQGYSVGSS LVEEDKLDLA TSLMEKAKTK GVSLLLPTDV VIADKFAADA NSKTVPASEI
GAQ85336.1  ---------- ---------- ---------- ---------- ---------- ---------- ---------- ---------- ----------
A8JFT4_PGK  ---------- ---------- ---------- ---------- ---------- ---------- ---------- ---------- ----------
```

```
              991
Zm00001d04  ---------- ---------- ---------- ---------- ---------- ---------- ---------- ---------- ----------
AQK68740.1  ---------- ---------- ---------- ---------- ---------- ---------- ---------- ---------- ----------
At_PGK3     ---------- ---------- ---------- ---------- ---------- ---------- ---------- ---------- ----------
Alyrata_PG  ---------- ---------- ---------- ---------- ---------- ---------- ---------- ---------- ----------
XP_0091066  ---------- ---------- ---------- ---------- ---------- ---------- ---------- ---------- ----------
XP_0137269  ---------- ---------- ---------- ---------- ---------- ---------- ---------- ---------- ----------
XP_0225516  ---------- ---------- ---------- ---------- ---------- ---------- ---------- ---------- ----------
XP_0091283  ---------- ---------- ---------- ---------- ---------- ---------- ---------- ---------- ----------
XP_0084470  ---------- ---------- ---------- ---------- ---------- ---------- ---------- ---------- ----------
NP_0013123  ---------- ---------- ---------- ---------- ---------- ---------- ---------- ---------- ----------
NP_0012754  ---------- ---------- ---------- ---------- ---------- ---------- ---------- ---------- ----------
NP_0013165  ---------- ---------- ---------- ---------- ---------- ---------- ---------- ---------- ----------
XP_0219934  ---------- ---------- ---------- ---------- ---------- ---------- ---------- ---------- ----------
XP_0220396  ---------- ---------- ---------- ---------- ---------- ---------- ---------- ---------- ----------
at_cPGK2    ---------- ---------- ---------- ---------- ---------- ---------- ---------- ---------- ----------
Alyrata_cP  ---------- ---------- ---------- ---------- ---------- ---------- ---------- ---------- ----------
At_cPGK1    ---------- ---------- ---------- ---------- ---------- ---------- ---------- ---------- ----------
Alyr_cPGK1  ---------- ---------- ---------- ---------- ---------- ---------- ---------- ---------- ----------
XP_0136482  ---------- ---------- ---------- ---------- ---------- ---------- ---------- ---------- ----------
XP_0091352  ---------- ---------- ---------- ---------- ---------- ---------- ---------- ---------- ----------
XP_0225670  ---------- ---------- ---------- ---------- ---------- ---------- ---------- ---------- ----------
XP_0136731  ---------- ---------- ---------- ---------- ---------- ---------- ---------- ---------- ----------
XP_0091246  ---------- ---------- ---------- ---------- ---------- ---------- ---------- ---------- ----------
XP_0136856  ---------- ---------- ---------- ---------- ---------- ---------- ---------- ---------- ----------
XP_0091465  ---------- ---------- ---------- ---------- ---------- ---------- ---------- ---------- ----------
XP_0084563  ---------- ---------- ---------- ---------- ---------- ---------- ---------- ---------- ----------
XP_0165815  ---------- ---------- ---------- ---------- ---------- ---------- ---------- ---------- ----------
PHT77520.1  ---------- ---------- ---------- ---------- ---------- ---------- ---------- ---------- ----------
XP_0042439  ---------- ---------- ---------- ---------- ---------- ---------- ---------- ---------- ----------
NP_0012752  ---------- ---------- ---------- ---------- ---------- ---------- ---------- ---------- ----------
XP_0151700  ---------- ---------- ---------- ---------- ---------- ---------- ---------- ---------- ----------
ADR71054.1  ---------- ---------- ---------- ---------- ---------- ---------- ---------- ---------- ----------
XP_0164941  ---------- ---------- ---------- ---------- ---------- ---------- ---------- ---------- ----------
XP_0220015  ---------- ---------- ---------- ---------- ---------- ---------- ---------- ---------- ----------
AQK86959.1  ---------- ---------- ---------- ---------- ---------- ---------- ---------- ---------- ----------
Zm00001d01  ---------- ---------- ---------- ---------- ---------- ---------- ---------- ---------- ----------
A8JC04_cPG  ---------- ---------- ---------- ---------- ---------- ---------- ---------- ---------- ----------
GAQ79801.1  ---------- ---------- ---------- ---------- ---------- ---------- ---------- ---------- ----------
sp_P18912.  ---------- ---------- ---------- ---------- ---------- ---------- ---------- ---------- ----------
AAC75963.1  ---------- ---------- ---------- ---------- ---------- ---------- ---------- ---------- ----------
sp|P50315|  ---------- ---------- ---------- ---------- ---------- ---------- ---------- ---------- ----------
sp_P00560.  ---------- ---------- ---------- ---------- ---------- ---------- ---------- ---------- ----------
NP_112467.  ---------- ---------- ---------- ---------- ---------- ---------- ---------- ---------- ----------
sp|P07205.  ---------- ---------- ---------- ---------- ---------- ---------- ---------- ---------- ----------
sp|P00558.  ---------- ---------- ---------- ---------- ---------- ---------- ---------- ---------- ----------
NP_032854.  ---------- ---------- ---------- ---------- ---------- ---------- ---------- ---------- ----------
Zm00001d03  ---------- ---------- ---------- ---------- ---------- ---------- ---------- ---------- ----------
tr_A0A1S4D  ---------- ---------- ---------- ---------- ---------- ---------- ---------- ---------- ----------
XP_0165427  ---------- ---------- ---------- ---------- ---------- ---------- ---------- ---------- ----------
XP_0063543  ---------- ---------- ---------- ---------- ---------- ---------- ---------- ---------- ----------
XP_0258888  ---------- ---------- ---------- ---------- ---------- ---------- ---------- ---------- ----------
XP_0042466  ---------- ---------- ---------- ---------- ---------- ---------- ---------- ---------- ----------
KAF3623713  PDGWMGLDIG PDAIKSFGSA LDSTKTIIWN GPMGVFEFDK FAAGTEAIAR KLAELSGKGV TTIIGGGDSV AAVEKVGLAD KMSHISTGGG
GAQ85336.1  ---------- ---------- ---------- ---------- ---------- ---------- ---------- ---------- ----------
A8JFT4_PGK  ---------- ---------- ---------- ---------- ---------- ---------- ---------- ---------- ----------
```

```
            1081
Zm00001d04  ---------- ----------
AQK68740.1  ---------- ----------
At_PGK3     ---------- ----------
Alyrata_PG  ---------- ----------
XP_0091066  ---------- ----------
XP_0137269  ---------- ----------
XP_0225516  ---------- ----------
XP_0091283  ---------- ----------
XP_0084470  ---------- ----------
NP_0013123  ---------- ----------
NP_0012754  ---------- ----------
NP_0013165  ---------- ----------
XP_0219934  ---------- ----------
XP_0220396  ---------- ----------
at_cPGK2    ---------- ----------
Alyrata_cP  ---------- ----------
At_cPGK1    ---------- ----------
Alyr_cPGK1  ---------- ----------
XP_0136482  ---------- ----------
XP_0091352  ---------- ----------
XP_0225670  ---------- ----------
XP_0136731  ---------- ----------
XP_0091246  ---------- ----------
XP_0136856  ---------- ----------
XP_0091465  ---------- ----------
XP_0084563  ---------- ----------
XP_0165815  ---------- ----------
PHT77520.1  ---------- ----------
XP_0042439  ---------- ----------
NP_0012752  ---------- ----------
XP_0151700  ---------- ----------
ADR71054.1  ---------- ----------
XP_0164941  ---------- ----------
XP_0220015  ---------- ----------
AQK86959.1  ---------- ----------
Zm00001d01  ---------- ----------
A8JC04_cPG  ---------- ----------
GAQ79801.1  ---------- ----------
sp_P18912.  ---------- ----------
AAC75963.1  ---------- ----------
sp|P50315|  ---------- ----------
sp_P00560.  ---------- ----------
NP_112467.  ---------- ----------
sp|P07205.  ---------- ----------
sp|P00558.  ---------- ----------
NP_032854.  ---------- ----------
Zm00001d03  ---------- ----------
tr_A0A1S4D  ---------- ----------
XP_0165427  ---------- ----------
XP_0063543  ---------- ----------
XP_0258888  ---------- ----------
XP_0042466  ---------- ----------
KAF3623713  ASLELLEGKQ LPGVLALDDA
GAQ85336.1  ---------- ----------
A8JFT4_PGK  ---------- ----------
```
